# Super-resolution imaging unveils the self-replication of tau aggregates upon seeding

**DOI:** 10.1016/j.celrep.2023.112725

**Published:** 2023-07-01

**Authors:** Eleni Dimou, Taxiarchis Katsinelos, Georg Meisl, Benjamin J. Tuck, Sophie Keeling, Annabel E. Smith, Eric Hidari, Jeff Y.L. Lam, Melanie Burke, Sofia Lövestam, Rohan T. Ranasinghe, William A. McEwan, David Klenerman

**Affiliations:** 1 Department of Chemistry, University of Cambridge, Lensfield Road, Cambridge CB2 1EW, UK; 2 UK Dementia Research Institute at University of Cambridge, Department of Clinical Neurosciences, Hills Road, Cambridge CB2 0AH, UK; 3 MRC Laboratory of Molecular Biology, Francis Crick Avenue, Cambridge CB2 0QH, UK

## Abstract

Tau is a soluble protein interacting with tubulin to stabilize microtubules. However, under pathological conditions, it becomes hyperphosphorylated and aggregates, a process that can be induced by treating cells with exogenously added tau fibrils. Here, we employ single-molecule localization microscopy to resolve the aggregate species formed in early stages of seeded tau aggregation. We report that entry of sufficient tau assemblies into the cytosol induces the self-replication of small tau aggregates, with a doubling time of 5 h inside HEK cells and 1 day in murine primary neurons, which then grow into fibrils. Seeding occurs in the vicinity of the microtubule cytoskeleton, is accelerated by the proteasome, and results in release of small assemblies into the media. In the absence of seeding, cells still spontaneously form small aggregates at lower levels. Overall, our work provides a quantitative picture of the early stages of templated seeded tau aggregation in cells.

## Graphical abstract

**Graphic abstract:**
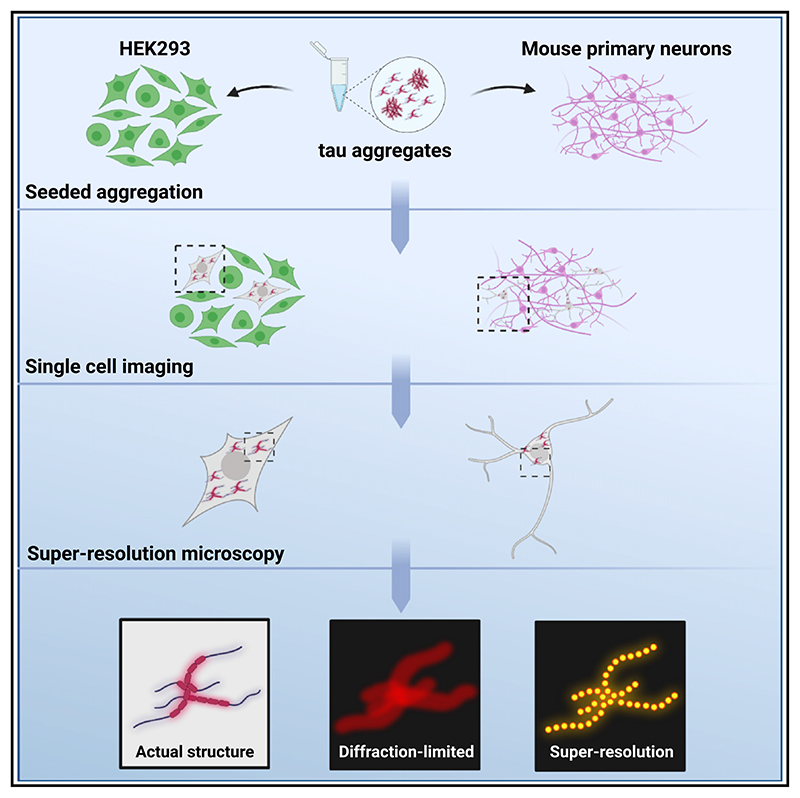

## Introduction

Dementia is one of the major causes of disability among older people. Worldwide approximately 50 million people have been diagnosed with the disease.^1^ Alzheimer’s disease (AD) is the most common form of dementia, characterized by extensive neuronal loss and severe brain shrinkage. This pathology is driven by the accumulation of extracellular amyloid-beta (Aβ) plaques and intracellular hyperphosphorylated tau aggregates.^2^ However, only the stereotypic development of tau pathology throughout the brain correlates well with the patient’s symptoms and the disease progression.^3^

Normally tau is a soluble protein interacting with tubulin to stabilize microtubules.^4^ This can be reversed by phosphorylation, leading to detachment of the protein from microtubules. Under pathological conditions, tau becomes hyperphosphorylated and aggregates to form fibrils, which play a pivotal role in AD pathogenesis.^5^ Determining the detailed molecular mechanisms involved in the development of tau pathology throughout the human brain is both fundamentally and therapeutically of great importance. However, the study of these aggregated species remains challenging, as they tend to be in low abundance and highly heterogeneous in terms of size and structure.

The aggregation of full-length tau is not readily induced in the test tube when compared to Aβ or α-synuclein,^6,7^ and usually cofactors such as heparin need to be present to trigger aggregation.^8^ Alternatively, *ex vivo*-derived pathological assemblies can be employed *in vitro* as seeds to trigger the aggregation of monomeric tau in a prion-like manner.^9,10^ The templated seeded aggregates can grow by addition of tau monomer into longer fibrils which can then self-replicate, for example by fragmentation into two daughter fibrils.^11^ Experimental evidence also suggests that seeds can spread transcellularly to neighboring cells and initiate a prion-like spreading process.^12,13^ However, our recent analysis of data from patients with AD demonstrated that the doubling time of tau aggregates within a given brain region is fairly slow (approximately 5 years) and that this replication timescale limits the overall speed of disease progression.^14^ Thus, the contribution of tau transcellular propagation in disease pathogenesis as well as the molecular mechanisms that govern this process remain broadly elusive.

The addition of fibrillar assemblies to cells in culture can induce intracellular tau aggregation,^15–17^ thereby providing a reproducible method to study the key steps of templated seeded tau aggregation in cells. However, these intracellular tau aggregates are structurally heterogeneous, typically smaller than the diffraction limit of visible light (~250 nm), and drastically outnumbered by monomers, thereby preventing the performance of quantitative experiments in intact cells with high resolution.

In the present study, we aimed to obtain a detailed nanoscopic characterization of the assemblies that are formed during the templated seeded aggregation of tau inside cells. To achieve this, we employed well-established antibodies against pathological tau assemblies and combined them with single-molecule localization microscopy.^18,19^ We found that initial formation of tau fibril-like assemblies takes place in close proximity to the microtubules. Small globular aggregates form as early as 4 h after seeding, before fibrillar-shaped aggregates are generated. Small aggregated species also form spontaneously in non-seeded primary neurons, suggesting that spontaneous tau aggregation can occur in the cellular environment. Finally, we provide evidence that inhibition of the proteasome (but not of autophagy) decelerates the amplification rate in the templated seeded aggregation of tau. Overall, our results show that tau aggregation proceeds rapidly in a cellular environment. The critical step appears to be the initial formation of aggregates in the cytosol, whose rapid growth and replication can then overcome the protective cellular machinery.

## Results

### Super-resolution imaging of tau aggregates in cells

To initially validate the antibodies that we used for tau imaging, we employed a HEK293 cell line that stably expresses the 0N4R isoform of human tau, bearing the frontotemporal dementia-associated mutation P301S, with a C-terminal Venus tag.^15^ We aimed to induce the aggregation of tau intracellularly by treating the cells with sonicated heparin-assembled recombinant P301S tau fibrils (Figure S1A) in the presence of lipofectamine. As a first step we characterized the recombinantly produced aggregates by assessing their entry to the cytosol and their seeding potency. By immobilizing the recombinantly produced assemblies on a glass surface and employing superresolution (SR) microscopy (Figure S1B), we calculated that approximately 25,000 assemblies with a mean length of 176 nm (Figure S1C) are added per cell when a concentration of 100 nM is employed. However, using our established tau entry assay,^20^ we estimated that only 2.5% of the added material reaches the cytosol of the HEK cells in 24 h (Figure S1D). Moreover, we observed that about 20% of the cellular population is seeded, as detected by the Venus fluorescence (Figures S1E and S1F). This process was tau specific and was not induced by other types of aggregates (Figure S1G). These results suggest that the entry of hundreds of tau seeds is needed to overcome the cellular protective mechanisms and induce intracellular tau aggregation.

**Figure 1:**
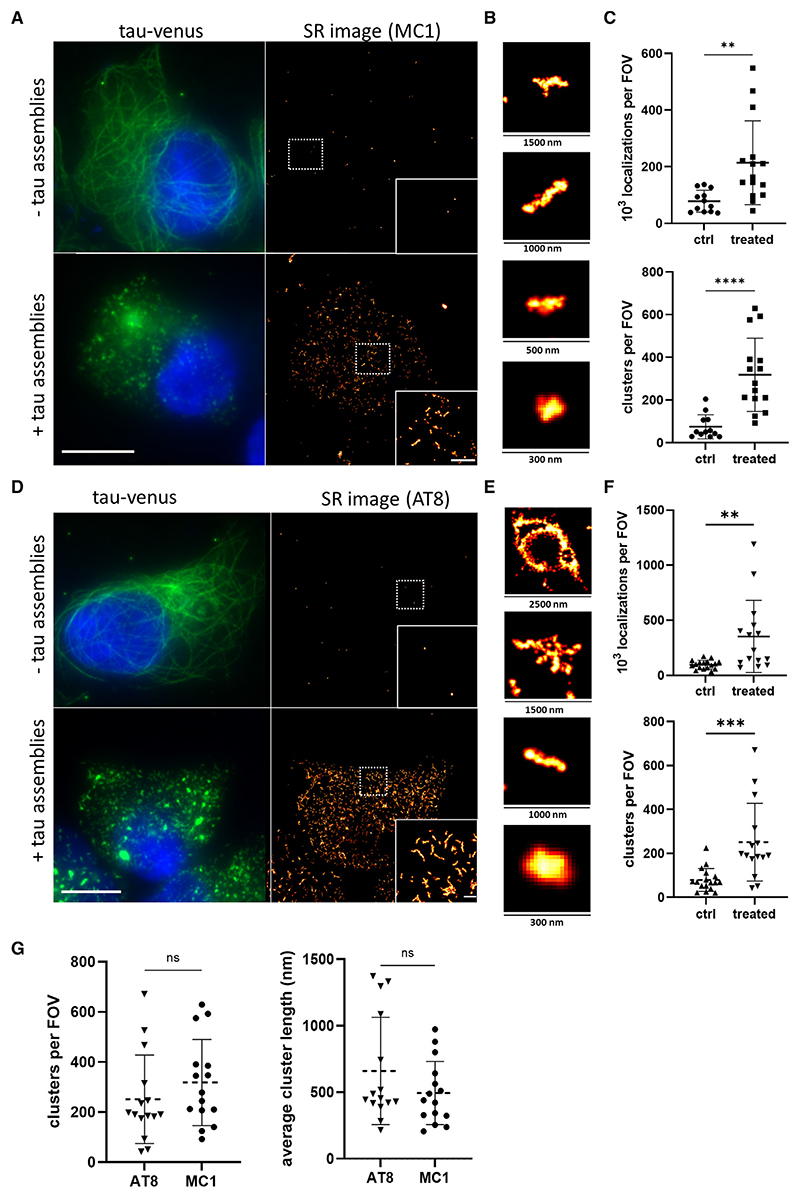
SR imaging of tau aggregates in HEK293 cells (A) Diffraction-limited (left) and SR (right) images of HEK293 cells stably expressing P301S tau-Venus with and without 100 nM recombinant P301S tau fibrils treatment for 24 h. Fixed cells were imaged using Venus fluorescence (green) for the imaging of total tau, while tau aggregates were visualized by STORM using the MC1 antibody (hot-red). Scale bars, 10 μm (left) and 2 μm (right). (B) Examples of individual tau aggregates of different sizes. (C) Cells treated with tau assemblies were compared to control cells for the number of detected localizations and the number of detected tau assemblies. (D) Diffraction-limited (left) and SR (right) images of HEK293 cells expressing P301S tau-Venus with and without 100 nM recombinant P301S tau fibrils treatment for 24 h. Fixed cells were imaged using Venus fluorescence (green) for the imaging of total tau, while tau aggregates were visualized by STORM microscopy using the AT8 antibody (hot-red). Scale bars, 10 μm (left) and 2 μm (right). (E) Examples of individual tau aggregates of different sizes. (F) Cells treated with tau assemblies were compared to control cells for the number of detected localizations and the number of detected tau assemblies. (G) Comparison of the number of detected tau assemblies (derived from C and F) and their length as detected by the MC1 and the AT8 antibody. The plotted data represent mean values ±SD. An unpaired t test was used for statistical analysis (n.s., not significant; **p < 0.01, ***p < 0.001, ****p < 0.0001) (n > 15 cells per condition were imaged from three biological replicates). See also Figures S1-S3.

We then employed the MC1 antibody, which specifically recognizes abnormal tau conformations,^21^ and combined it with direct stochastic optical reconstruction microscopy (dSTORM)^22^ to image the intracellularly formed tau assemblies with spatial resolution of 38 nm and localization precision of ~16 nm (Figures 1A and 1B). We were also able to detect very low levels of MC1 immunoreactivity in the untreated condition, which is potentially derived from either spontaneously formed tau aggregates or background signal from the antibody (Figure 1A). We further quantified the number of positional localizations of individual active fluorophores and identified clustered tau molecules that would indicate the formation of aggregates. The number of detected localizations per field of view (FOV) was much higher for treated than control cells (on average 214 and 78 localizations, respectively), which resulted in a highly increased number of aggregates for treated cells (Figure 1C). The intracellularly formed tau aggregates were also super-resolved using the AT8 antibody, which specifically recognizes phosphorylated tau at Ser202/ Thr205 (Figures 1D–1F). This antibody specifically recognized newly formed tau assemblies inside cells and, in contrast to MC1, it does not detect the added recombinant tau seeds because the latter lack post-translational modifications. The imaging revealed the presence of intracellular assemblies similar in terms of shape and size to the ones detected by the MC1 antibody (Figure 1G). Overall, these results confirm the suitability of both antibodies for the nanoscale characterization of seeded tau assemblies in cells.

We further aimed to expand the characterization of the intracellularly produced seeded assemblies. To achieve this, we biochemically isolated the Sarkosyl-insoluble species produced 24 h after seeding in HEK293 cells expressing tau P301S-Venus. The extracted material was assessed by negative-stain electron microscopy as well as by immunogold labeling with the AT8 phospho-specific antibody and gold-conjugated nickel-nitrilotriacetic acid (Ni-NTA) particles. We were able to detect fibrillar species that were distinguishably wider in diameter when compared to the exogenously administered recombinant material (~16 nm diameter for the recombinant and ~32 nm for the tau P301S-Venus) (Figure S1I), a feature most likely attributed to the presence of the fluorescent tag.^23^ Notably, we were not able to detect any traces of the recombinant fibrils incorporated within the cell-derived filaments but rather a homogeneous population of wider fibrillar species (Figure S1H). Furthermore, and in line with our dSTORM data, we observed strong immunogold labeling with the phospho-specific AT8 antibody for most of the cell-extracted fibrils (Figure S1J), while the absence of positive labeling with gold Ni-NTA particles on the cell-derived filaments (Figure S1J) strongly indicates the presence of *de novo* generated aggregates that solely consist of the endogenously expressed tau. Finally, the cell-derived assemblies were employed as seeding material for secondary inoculation into tau-expressing cells and demonstrated significant seeding competency (Figure S1K), collectively suggesting the amplification of intracellular tau aggregates as a result of templated seeded aggregation reactions.

As a next step, we employed a recently established method, called Exchange PAINT (point accumulation for imaging in nanoscale topography),^24^ to study the subcellular location at which tau aggregation is initiated after seeding. Using antibodies that are linked with different DNA-PAINT docking strands, this methodology enables multiplexed SR imaging of different cellular components by using the same fluorescence channel after exchanging different complementary imager strands, which are labeled with the same fluorescent dye. In line with our dSTORM analysis, we detected AT8-postive tau assemblies after seeding. Interestingly, the resulting SR images showed clear co-localization of the newly formed aggregates with the microtubules (Figure S2A). More specifically, in the early stages of seeded aggregation, small globular aggregates were apparent on the microtubules (Figure S2A, a and b). In cells with higher number of formed assemblies mature fibrillike structures were detectable, which appear to interact with the microtubule cytoskeleton (Figure S2A, c and d). These findings were further supported by diffraction-limited live-cell imaging experiments following the Venus fluorescence (Video S1), in which the tau assemblies did not freely diffuse in the cytosol but were fairly immobile and co-localized with the cytoskeleton. To test whether the association of tau with the microtubules is essential for the initiation of seeded tau aggregation, we treated the cells with nocodazole, which interferes with the polymerization of microtubules. Indeed, upon treatment with the compound, the microtubules depolymerized and tau dissociated from the cytoskeleton (Figure S2B). We further detected increased levels of aggregation when the cells were treated with tau aggregates in the presence of nocodazole (Figure S2C). Collectively, these data suggest that seeded aggregation is more efficient when tau is free in the cytosol. However, since the majority of tau is associated with the microtubules, seeded tau aggregation might occur in close proximity to the cytoskeleton, from where seeds can potentially recruit tau monomers during elongation.

### P301S tau aggregates replicate rapidly after seeding in cells

Recent studies claimed that fluorescent tagging of tau interferes with the templated seeded aggregation properties in biosensor cell lines.^23^ To ensure that in our experimental setup the Venus tag does not influence the characteristics of tau aggregates, we generated a HEK293 cell line stably expressing untagged 0N4R P301S tau for direct comparison. Treatment of both cell lines with recombinant tau fibrils under the same experimental conditions led to the formation of intracellular tau assemblies that were similar in shape and localization (Figures S3A and S3B). We also compared the number of assemblies in our newly created cell line with control HEK293 cells to determine the AT8 background signal as well as to test whether spontaneously formed tau assemblies are present. We detected a low number of assemblies in both cell lines, which is presumably due to non-specific binding of the antibodies, while no spontaneously formed assemblies were detected in untreated P301S tau-expressing cells (Figure S3C).

**Figure 2:**
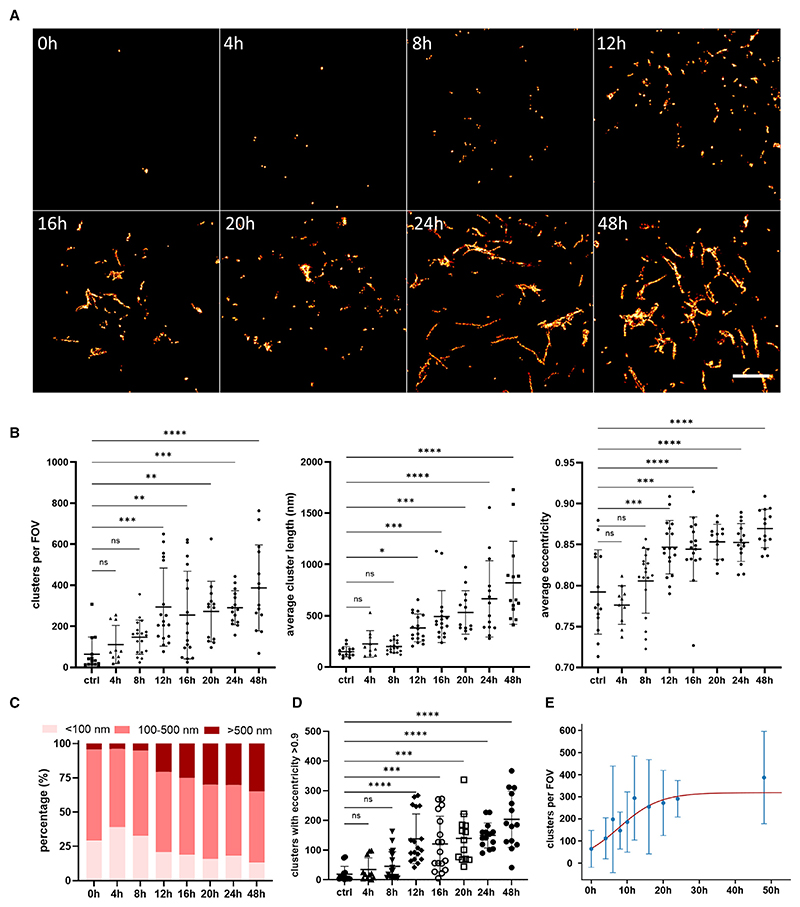
SR images reveal time-dependent replication of endogenous tau assemblies after treatment with tau assemblies (A) HEK293 cells expressing untagged P301S tau were treated with 100 nM recombinant P301S tau fibrils. At defined time points, cells were fixed and immunostained with the AT8 antibody for dSTORM imaging. Representative SR images of a zoomed area in a cell are displayed. Scale bar, 3 μm. (B) The number of assemblies detected per FOV, as well as their length and average eccentricity, were analyzed and plotted. (C) The percentage of aggregates with length less than 100 nm or more than 500 nm was quantified. (D) The number of tau assemblies with an eccentricity higher than 0.9 was plotted. (E) Kineticanalysisoftheformation ofintracellularaggregates. Dataare shown as meanvalues(±SD) from (B), but all data points are used inthe fitting toaminimal model of replication. The statistical analysiswas based on a one-wayANOVAtest combined with Tukey’s post hoctest (n.s., not significant; *p < 0.05, **p < 0.01, ***p < 0.001, ****p < 0.0001) (n > 10 cells per condition were imaged from three biological replicates). See also Figures S3 and S4; Table S1.

As a next step, we aimed to study in a time-dependent manner the templated seeded aggregation of tau in intact cells. To achieve this, we employed our newly developed cell line expressing the untagged P301S tau and acquired SR images of individual cells at defined time points for 48 h. The data revealed the rapid elongation of tau aggregates within 12 h after the addition of seeds (Figures 2A and 2B). The SR images enabled the detailed characterization of the newly formed aggregates and the study of their length and eccentricity (Figures 2B and S4A–S4C). The average length of the assemblies at the 24 h time point was calculated to be 660 nm. Interestingly, the proportion of small assemblies (<100 nm) rapidly increased within 4 h after seeding but decreased in later stages that were characterized by the concomitant formation of longer tau assemblies (>500 nm) (Figures 2C and S4C). We quantified the amount of fibril-like tau structures as defined by an eccentricity higher than 0.9,^25^ and we observed a steep increase in the number of fibrils between the 8 and 12 h time points (Figure 2D), indicating that once the seeded aggregation is initiated, the formation of tau fibrils can be induced rapidly. Kinetic analysis of the data further showed the self-replication of tau aggregates with an initial doubling time of approximately 5 h, followed by a plateauing phase around 1 day after the addition of the seeds (Figure 2E).

**Figure 3:**
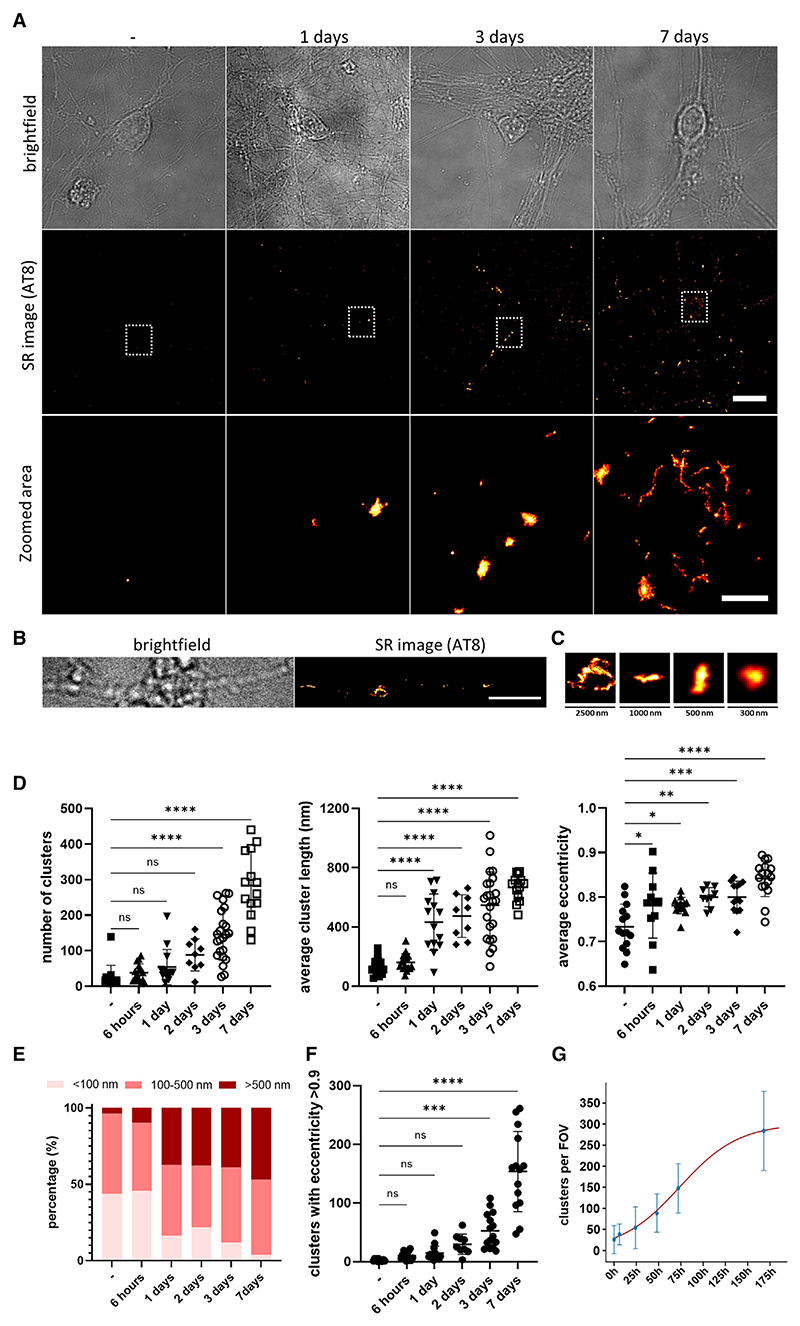
Intracellular tau assemblies are formed upon treatment of primary cultures with recombinantly produced tau fibrils (A) DIV 7 primary cultures derived from P301S tau transgenic mice were treated with 100 nM recombinantly produced tau fibrils. At defined time points, the cultures were fixed and immunostained with the AT8 antibody for dSTORM imaging. Scale bars, 10 μm (top) and 2 μm (bottom). (B) Representative bright-field and SR images of tau aggregates as detected in neuronal processes 3 days after treatment. Scale bar, 5 μm. (C) Representative examples of individual tau aggregates of different sizes from (B). (D) The number of assemblies detected per FOV as well as their lengths and average eccentricity were analyzed and plotted. (E) The percentage of aggregates with length less than 100 nm or more than 500 nm was quantified. (F) The number of tau assemblies with an eccentricity higher than 0.9 was plotted. (G) Kinetic analysis of the formation of intracellular aggregates. Data are shown as mean values (±SD) from(D),but all data points are used in the fitting to a minimal model of replication. The statistical analysis was based on a one-way ANOVA test combined with Tukey’s post hoc test (n.s., not significant; *p < 0.05,**p < 0.01, ***p < 0.001, ****p < 0.0001) (n > 9 FOVs per condition were imaged from three biological replicates). See also Figure S5 and Table S1.

We then extended our study of the kinetics of seeded tau aggregation to primary neurons derived from P301S tau transgenic mice.^26^ In line with previous reports,^20^ we found that even in the absence of lipofectamine, ~7.5% of the added recombinant tau aggregates entered the cytosol of neurons in the first 24 h (Figure S5A) and were able to induce widespread aggregation of intracellular tau pools. We next treated the culture after 7 days *in vitro* (DIV) with 100 nM recombinant tau fibrils and followed the formation of intracellular tau aggregates over time (Figure 3A). Using our SR experimental approach, we already detected an increase in the number of AT8-positive tau assemblies 24 h after seeding, and this significantly increased over 3 days (Figure 3D). Interestingly, we found that tau aggregation was initiated in neuronal processes, while soma-localized aggregates were detected in small amounts after 3 days but significantly increased after 7 days (Figures 3A–3C and S5). The size and shape of the formed assemblies was similar to the ones observed in HEK cells, indicating that the cell type does not influence the shape of the formed assemblies upon templated seeded aggregation (Figure 3D). Intriguingly, we found that the average size and eccentricity of the aggregates already significantly increased 24 h after seeding (Figures 3D–3F and S5C), suggesting the formation of intracellular fibrillar aggregates after the treatment. In support of this observation, the assemblies formed were positive for staining with the amyloid-specific dye pFTAA^27^ and the conformation-specific antibody MC1 (Figure S5D). Indeed, kinetic analysis revealed a doubling time of approximately 1 day in primary murine neuronal cultures, which is significantly longer than the 5 h observed in HEK cells (Figure 3G), despite the higher levels of tau expression in the primary neurons (Figure S5E).

**Figure 4:**
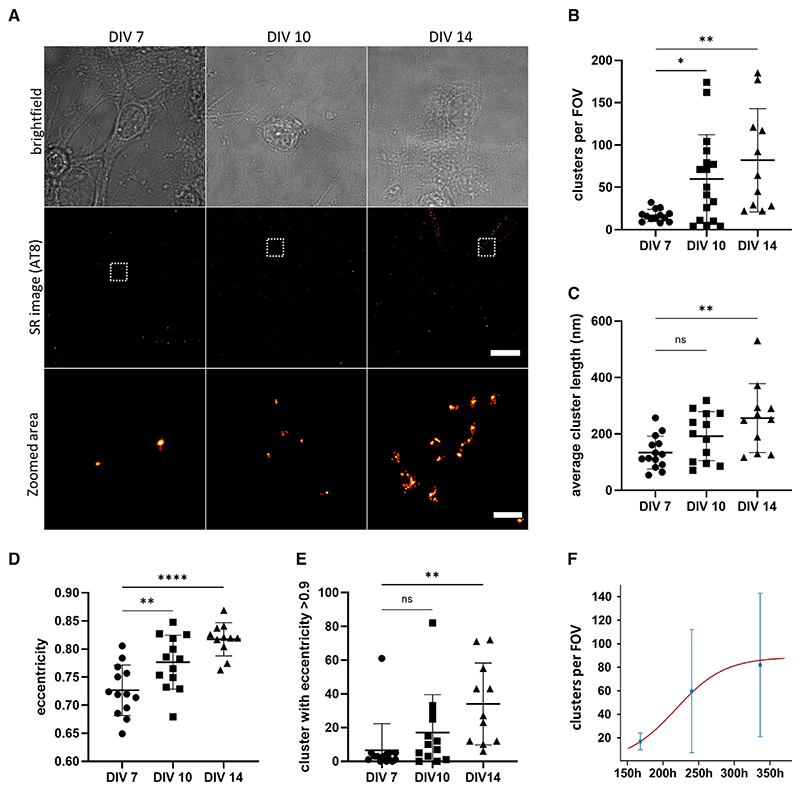
Small tau assemblies are formed spontaneously in primary cultures derived from P301S tau transgenic mice (A) Untreated DIV 7, 10, and 14 primary cultures derived from P301S tau transgenic mice were fixed and immunostained with the AT8 antibody for dSTORM imaging. Scale bars, 10 μm (top) and 2 μm (bottom). (B–D) The number of assemblies detected per FOV (B), their length (C), and their eccentricity (D) were analyzed and plotted. (E) The number of assemblies with eccentricity higher than 0.9 were quantified. (F) Kinetic analysis of the spontaneous formation of intracellular aggregates over time; data are shown as mean values (±SD) from (B), but all data points are used in the fitting to a minimal model of replication. The statistical analysis was based on a one-way ANOVA test combined with Tukey’s post hoc test (n.s., not significant; *p < 0.05, **p < 0.01, ****p < 0.0001) (n > 11 FOVs per condition were imaged from three biological replicates). See also Table S1.

We also observed that small numbers of AT8-positive tau assemblies were formed in these cells spontaneously in the absence of seeding (Figures 4A and 4B). These clusters were quite small (Figure 4C) but their eccentricity increased over time, suggesting the formation of short fibrillar assemblies (Figures 4D and 4E). In contrast, the number of detected clusters due to non-specific binding of the AT8 antibody in control neurons that do not express human tau remained low and did not increase over time (Figure S5F). This indicates that in cultured primary neuronal cells expressing human P301S tau, the latter can undergo self-association over time in the absence of seeding. Interestingly, the doubling time for the spontaneous formation of tau aggregates was found to be 21 h, which is very close to the result obtained upon seeding, although this best fit value was only weakly constrained compared to the seeded case (Figures 3G and 4F; Table S1).

### Seeded aggregation of wild-type tau occurs at a slow rate

**Figure 5:**
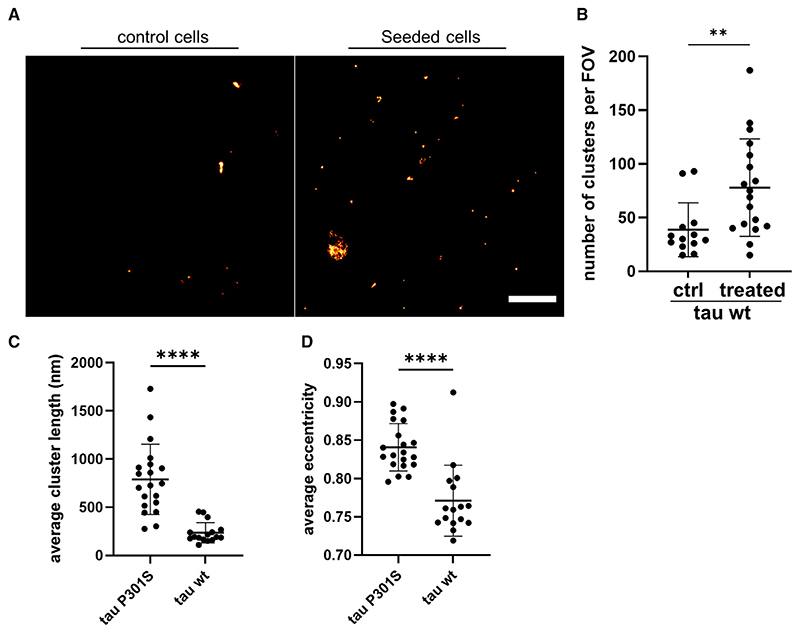
Templated seeding characteristics of WT tau in cells (A) HEK293 cells expressing untagged WT tau were imaged by dSTORM after treatment with 100 nM recombinant P301S tau fibrils for 24 h and subsequent immunolabeling with the AT8 antibody. Scale bar, 3 μm. (B) The number of formed assemblies were compared to mock-treated cells. (C and D) The average length of the formed clusters (C) as well as their eccentricity (D) were compared to cells expressing P301S tau after being treated under the same conditions. The plotted data represent mean values ±SD. The statistical analysis in (B), (C) and (D) was based on an unpaired t test (**p < 0.01, ****p < 0.0001) (n ≥ 13 cells per condition were imaged from three biological replicates).

A significant experimental challenge in the field has been the study of wild-type (WT) tau aggregation, as it aggregates at a very slow rate compared to the P301S variant.^11,12^ Further major hurdles in the study of WT tau seeded aggregation in cell-culture models are the low levels of aggregate formation and the limited resolution provided by conventional bulk biochemical techniques.^9^ To overcome these limitations, we generated a HEK293 cell line expressing untagged WT tau at levels comparable to those of the aforementioned cells expressing the P301S tau variant (Figure S6A) (intracellular tau concentration of both cell lines has been calculated to be approximately 300 nM). This newly generated cell line was treated with recombinant P301S tau fibrils and using our AT8-bound ultrasensitive SR microscopy approach on intact cells, we detected a small, but significant, increase in the number of intracellular tau aggregates (Figures 5A and 5B). Notably, this number of aggregates was approximately four times lower when compared to corresponding treatment of the P301S tau-expressing cells (Figure 2). Furthermore, the WT tau aggregates were smaller (230 nm for WT and 790 nm for P301S tau), more globular in shape, and demonstrated low eccentricity values when compared to the aggregates formed in P301S tau-expressing cells (Figures 5C and 5D).

**Figure 6:**
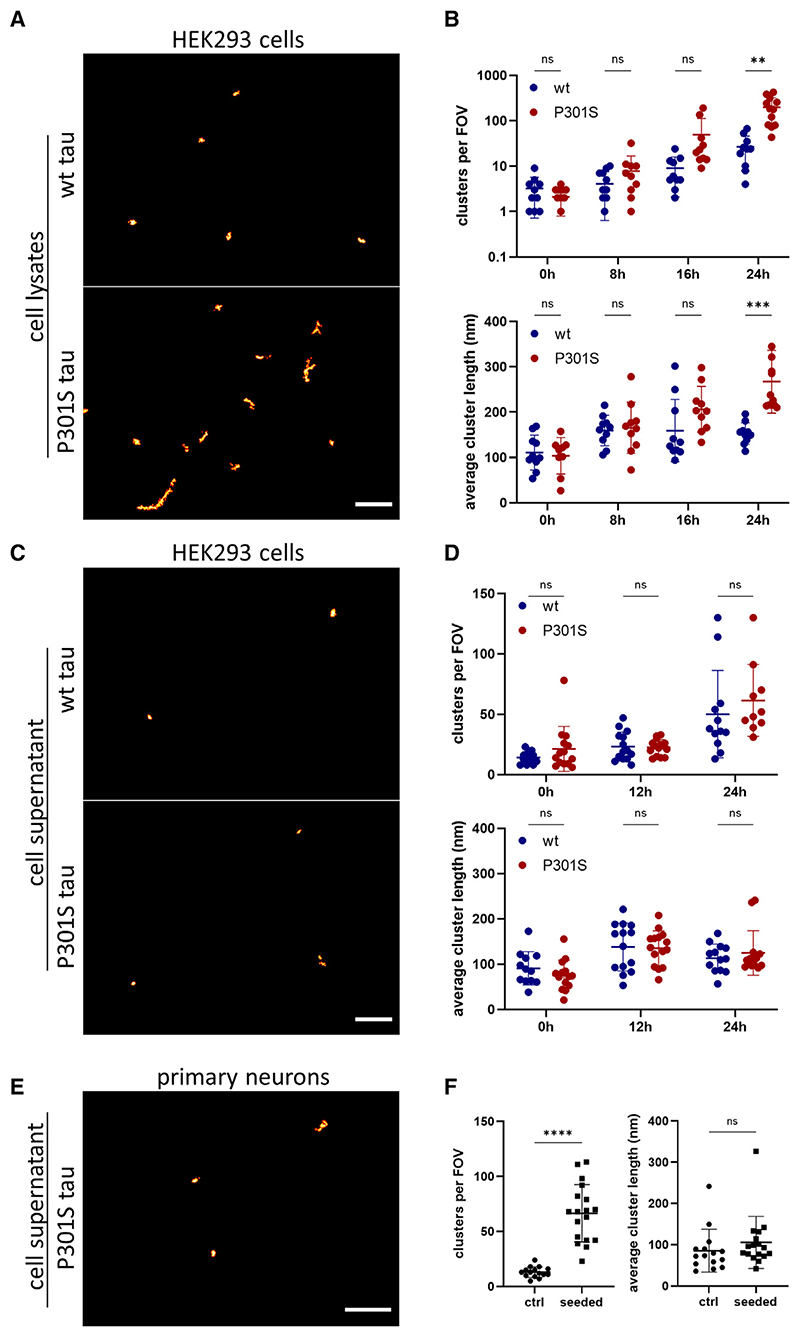
DNA-PAINT on lysates and media from cells expressing P301S tau or WT tau upon treatment with recombinant P301S tau fibrils (A-D) Representative images of HEK293 cell lysates (A) and cell supernatant (C) that were collected 24 h after treatment. The number of tau assemblies per FOV and their average length were followed over time and plotted for both lysates (B) and media (D). (E) Representative images of cell supernatant from primary neurons 7 days after treatment. (F) The number of tau assemblies per FOV and their average length plotted for control and seeded cells. Scale bars, 1 μm. The plotted data represent mean values of each experiment ±SD. The statistical analysis in (B) and (D) was based on a two-way ANOVA test, while an unpaired t test was performed for the data plotted in (F) (n.s., not significant; **p < 0.01, ***p < 0.001, ****p < 0.0001) (n = 3 biological replicates). See also Figure S6 and Table S1.

We then aimed to compare in more detail the seeded aggregation kinetics between these two tau variants. To achieve this, we adapted a previously reported single-molecule pull-down (SiMPull) assay.^28^ This method combines a conventional immunoprecipitation approach with single-molecule fluorescence imaging, thereby enabling the rapid and sensitive imaging of individual aggregated protein assemblies. More specifically, we employed a biotinylated version of the AT8 antibody to capture the phosphorylated tau and performed DNA-PAINT to superresolve the captured tau aggregates. Using this assay, we could specifically detect the aggregates in lysates from cells expressing P301S or WT tau 24 h after treatment with recombinant tau fibrils or monomer (Figures 6A and 6B). Similar to what we observed in intact cells, the number of aggregates in WT tau-expressing cells was lower and smaller in size than in the cells expressing P301S tau, although both increased in number and size over time (Figure 6B). An increase in the formation of tau aggregates was detectable for the WT tau as early as 16 h after seeding (Figure 6B), while this increase in phosphorylation could not be detected by conventional dot blot before the 48-h time point (Figure S6A). The doubling time for P301S tau was found to be 3 h, close to what was calculated in fixed cells, while WT tau aggregates were calculated to replicate more slowly, with a doubling time of 5 h (fits in Figures S6C and S6D). The small difference in the doubling time and size of the P301S tau aggregates between the SiMPull and the fixed-cell experiments may be due to dissociation of big clusters of fibrils upon cell lysis and to the higher sensitivity of the SiMPull assay.

Moreover, we examined the presence of tau assemblies in the extracellular space. Tau aggregates were released into the cell media after seeding (Figures 6C and 6D) and were very small, a finding in line with previous reports on secreted tau species.^13,29,30^ This increased release of non-fibrillartau aggregates might be a form of cell response to the disruption of protein homeostasis. However, it cannot be ruled out that this release is caused by cell death after treatment with recombinant aggregates, as suggested by the increased extracellular lactate dehydrogenase (LDH) levels (Figure S6B). We further examined the number and size of tau aggregates in the media of control and seeded murine primary neurons expressing the human P301S tau variant (Figures 6E and 6F). We found that a higher number of tau assemblies was present in the cell supernatant of seeded cells compared to control cells (Figure 6F). Interestingly, the shape and size of these tau aggregates was very similar to those of the aggregates found in the cell supernatant of seeded HEK cells. This indicates that primary neurons can also release small tau aggregates into the extracellular space.

### Seeded tau aggregation is accelerated by the proteasome

**Figure 7:**
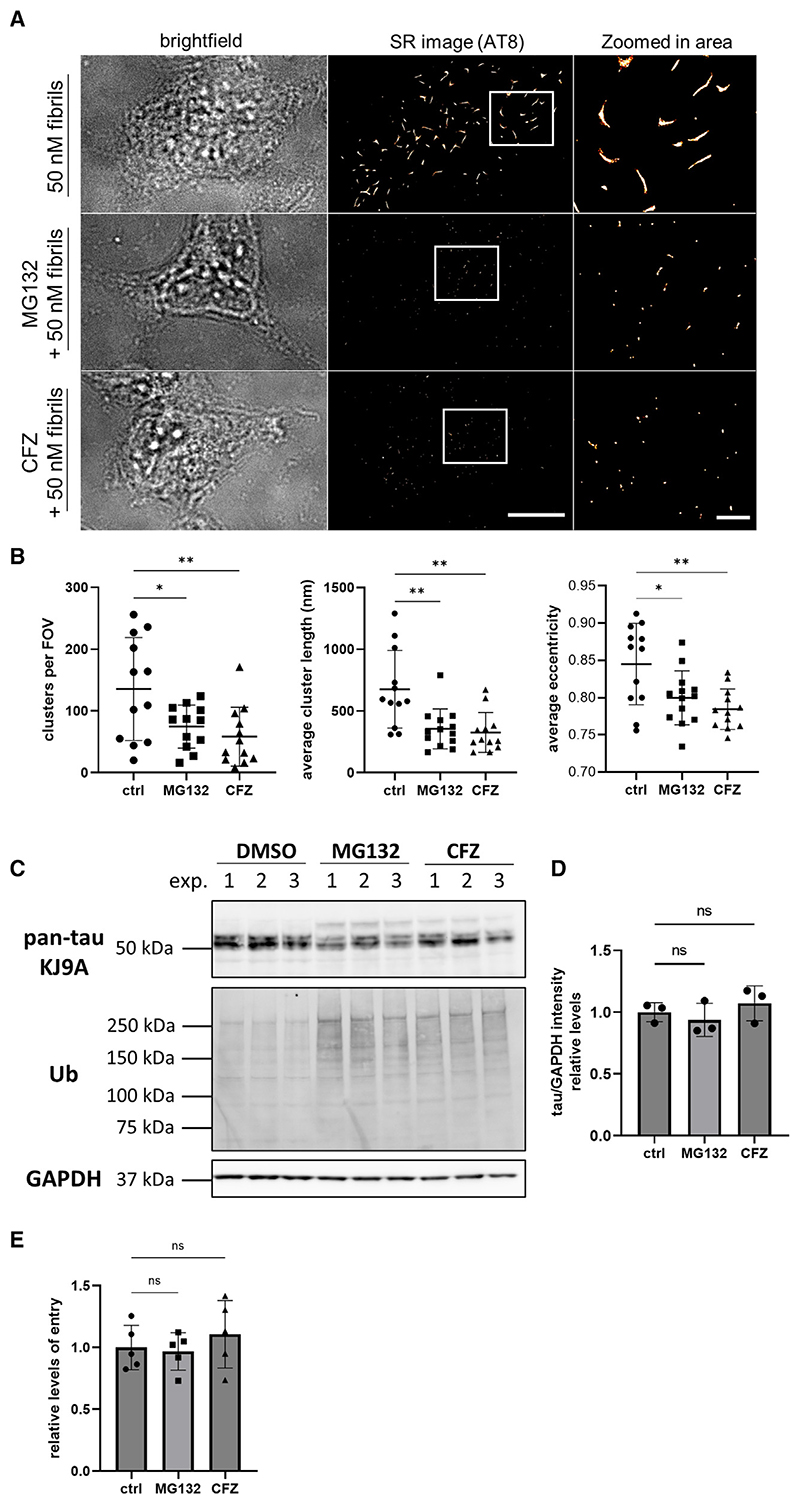
Proteasomal inhibition reduces the templated seeded aggregation of tau in HEK293 cells (A) Representative bright-field and AT8-STORM images of HEK293 cells expressing P301S tau treated with 50 nM recombinant P301S tau fibrils in the presence of MG132 or carfilzomib (CFZ) for 16 h. Scale bars, 10 μm (left) and 2 μm (right). (B) The number of aggregates per FOV, the average length, and eccentricity of the clusters were quantified (n R 13 cells per condition were imaged from three biological replicates). (C) Western blot analysis of lysates from HEK293 cells expressing untagged P301S tau in the presence of proteasome inhibitors. The cell lysates were assessed for intracellular tau levels via the pan-tau KJ9A antibody as well as for the levels of ubiquitinated proteins, while GAPDH was used as loading control. (D) Quantification of intracellular tau levels upon normalization to GAPDH and subsequent comparison to the untreated control (n = 3). (E) The entry levels of 100 nM tau-HiBiT assemblies in cells expressing NLS-eGFP-LgBiT in the presence of lipofectamine for 4 h and the corresponding proteasomal inhibitors were quantified and then compared to the untreated control (n = 3). The plotted data represent mean values ±SD. The statistical analysis is based on a one-way ANOVA test combined with Tukey’s post hoc test (n.s., not significant; *p < 0.05,**p < 0.01). See also Figure S7.

Next, we investigated the potential role of the proteasome in the seeded tau aggregation, as previous studies have reported that tau is degraded by the proteasome^31–33^ and the resulting species display severe toxicity to the cells.^34^ For this purpose, we performed seeding experiments in HEK293 cells expressing untagged P301S tau in the presence of two different proteasomal inhibitors, MG132 and carfilzomib (CFZ). Remarkably, 16 h after treatment, the number of AT8-positive tau assemblies per cell was reduced by almost 50% in the presence of proteasomal inhibitors (Figures 7A and 7B). This also affected the average size of the detected clusters, which was reduced by more than half and resulted in more globular aggregates, as indicated by the average eccentricity of the clusters (Figure 7B). This observation was not a result of reduced intracellular expression levels or impaired uptake of the exogenously added assemblies, as both remained unaffected upon treatment with the inhibitors (Figures 7C–7E). However, when we modulated the autophagy degradation pathway, either negatively by inhibition of autophagosome-lysosomal fusion with bafilomycin A1 or positively by rapamycin-mediated inhibition of mTORC1, the number, length, and eccentricity of the formed tau assemblies upon seeding remained unaffected (Figure S7). Collectively, these results support the importance of the proteasomal pathway during the amplification of pathological tau species in cells and demonstrate the applicability of our newly developed nanoscale methods in the detailed characterization of templated seeded tau aggregation.

## Discussion

Over the last 15 years a variety of cell-culture models expressing fluorescently labeled tau fragments or full-length mutant variants have been generated to study the process of templated seeded aggregation of tau.^15,16,35,36^ However, the conventional biochemical techniques that are commonly employed lack the necessary resolution and sensitivity for the detection and characterization of newly formed tau aggregates, which are in low abundance and highly heterogeneous in terms of size and structure.

In the current study, we used HEK293 cells and mouse primary neurons expressing WT or P301S tau to study the early stages of seeded as well as spontaneous tau aggregation at a single-aggregate resolution. This significant improvement of resolution enables the visualization of very small aggregates, which otherwise cannot be detected by diffraction-limited imaging techniques. More specifically, our SR imaging allowed us to study the kinetics and the subcellular localization of tau aggregation in cell-culture systems from very early stages. To this end, our Exchange PAINT SR microscopy experiments revealed that formation of tau aggregates after seeding occurred close to the cytoskeleton. Under physiological conditions, the intracellular concentrations of tubulin in living cells can be as high as 24 μM,^37,38^ and tau has a very high binding affinity to the microtubules via the repetitive regions that are located in the C-terminal domain.^39^ Based on the calculated tau concentration inside our newly developed HEK293 cells lines (~300 nM) and combined with previously reported K_D_ values between tau and microtubules at around 680 nM,^12,13^ we anticipate that the vast majority of the expressed tau will be either bound or in close proximity to the microtubules. This situation could potentially promote the templated seeding phenomena to occur in the vicinity of the microtubule cytoskeleton, where the local concentration of tau is high. Supportive of this, a previous study has also reported the spontaneous formation of oligomeric tau structures on the microtubules.^40^ Conversely, disruption of the microtubule network in our study significantly enhanced the intracellular seeding propensity, potentially due to the increased levels of free tau to “feed” the templated seeding reaction. Along the same lines, we observed that the seeded aggregation of tau in neurons is initiated in the neuronal processes, an area dense in microtubules and where soluble tau has been reported to localize in higher concentrations.^41,42^ In contrast to previous studies that report translocation of hyperphosphorylated tau to the soma before its aggregation,^42,43^ we observed tau clusters being present in the soma only in later stages when high numbers of tau aggregates are present inside the cell.

Prion-like seeded aggregation of tau requires the uptake of tau aggregates and their delivery to the cytosol, where aggregation of monomeric tau is induced. In our study, we initially characterized the entry and seeding potency of the recombinant tau seeds in the cell-culture systems employed. We found that a high number of aggregates is required to enter the cytosol to induce effective seeding. Using a previously established tau entry assay,^20^ we calculated that only small amounts of tau seeds enter the cytosol of HEK cells even in the presence of lipofectamine. More precisely, only 2.5% of the administered recombinant aggregates reached the cell cytosol, which translates into ~500 recombinant tau aggregates per cell for our experimental conditions. This calculation, combined with the observation that only 20% of the cellular population is seeded, suggests that a high number of tau aggregates need to access the cytosol to disrupt the cellular protective mechanisms and effectively induce tau aggregation. Similar observations have also been reported for experiments in organotypic hippocampal slice cultures from P301S tau transgenic mice,^44^ in which seeding was only observed when high concentrations (100 nM or more) of tau assemblies were administered. The concentration of tau in the interstitial and the cerebrospinal fluid of AD patients has been calculated to be at least 100 times lower,^44–46^ suggesting that increased levels of released tau locally might be required to achieve efficient spreading of the pathology. Interestingly, cytosolic entry of tau to primary neurons was found to be more efficient, with ~3 times higher entry levels compared to HEK cells despite the absence of lipofectamine during administration. Tau has been reported to enter neurons and HEK cells by different mechanisms,^20^ which could in turn explain the observed deviations. Interestingly, despite the higher entry potency of the recombinant tau assemblies and the higher intracellular expression levels of tau in primary neurons, templated seeded aggregation was slower than in HEK293 cells, potentially due to the presence of more effective neuronal protective mechanisms that act to limit tau aggregation.

While the templated seeded aggregation of tau in cells has been demonstrated previously by many groups,^15,16,35,36^ here we follow and quantify the self-replication of tau aggregates at earlier stages. We found that once the administered tau fibrils enter the cytosol, rapid formation of globular AT8-positive tau aggregates occurs, which then grow into longer fibrils that are structurally and immunoreactively distinct from the exogenously added species. The time required to double the number of aggregates was ~5 h for P301S tau in HEK cells and ~24 h for the primary neurons. In HEK cells small non-fibrillar aggregates form initially, already with a size range from 50 up to 800 nm at 8 h after treatment, which over longer time periods grow into longer fibrils, reaching a length of up to 8 μm in 48 h. Tau seeded aggregation was much slower in primary neurons than in HEK cells; however, long tau fibrils with an average length of 430 nm were already detectable 24 h after treatment with recombinant seeds. It is worth noting that the average length and number of clusters with fibril-like structure are comparable at the end of the seeding experiments for both cell types. However, the percentage of clusters with a length bigger than 500 nm in neurons is almost 50% while in HEK293 cells this is approximately 30%, a difference that could be explained by the longer incubation period post seeding. Interestingly, the doubling time for tau aggregates is approximately 8- and 2-fold faster in neurons and HEK293 cells, respectively, compared to the rate that has been reported *in vitro.*^11^ This observation can be attributed to post-translational modifications occurring concurrently with tau aggregation inside cells as well as to proteostasis pathways that may contribute by increasing the fragmentation of longer fibrils. One potential mechanism that has been previously reported involves the disassembly of tau fibrils by chaperones, which leads to the formation of smaller and more seeding-competent species.^47^ An alternative pathway involves the proteasome as a key player in accelerating tau fibril fragmentation.^34^ Consistent with this, tau fibrils supplied to the extracellular space have been shown to enter the cytosol and become targets for proteasome activity.^33^ Indeed, by blocking the proteasomal activity we were able to prevent this fast aggregate formation inside the cells. Notably, our studies have also shown that inhibition of the proteasome leads to a significant reduction of α-synuclein seeded aggregation in cells,^48^ indicating a common molecular mechanism between these two proteins.

Another important observation was that murine primary neurons expressing P301S tau can spontaneously form short AT8-positive clusters with high eccentricity, whose doubling rate seems to be comparable to that of the seeded condition (approximately 1 day). This result is consistent with the *de novo* assembly of tau aggregates without any exogenous administration of seeds in the original *in vivo* mouse model^26^ and these spontaneously formed tau filaments are detectable in the lumbar spinal cord within 1 month of age.^49^ Our findings suggest that even though seeded aggregation inside cells bypasses the primary nucleation in a similar manner to *in vitro* aggregation experiments,^9,50^ the doubling time of the newly formed aggregates remains unaffected and is mediated by other factors, such as the proteasomal fragmentation.^34^ Interestingly, the doubling time of seed-competent tau species in a different P301S tau model was estimated to be approximately 2 weeks,^14^ suggesting an *in vivo* rate of tau amplification ~14 times slower than in cultured cells. Moreover, our analysis of a recent study focusing on seeded aggregation of WT tau in human induced pluripotent stem cell (iPSC)-derived neurons^51^ estimated the doubling time to be ~2 weeks (Table S2). As an intriguing estimation, one can combine the 14-fold decrease between cell culture and *in vivo* with this 14-fold difference between WT and mutant tau in cell culture, collectively resulting in a doubling time of ~200 days *in vivo*. Although this is far less compared to the 5 years’ doubling time of aggregated tau that we previously determined in the brain of AD patients,^14^ it should be noted that the slower replication rates *in vivo* might be a result of supportive clearance mechanisms for toxic by-products.^52,53^

It is worth noting that the doubling times calculated *in vivo* are generally determined from measurements of entire brain regions and thus contain contributions from both the aggregate replication within a neuron and local intraneuronal spread. Thus, the low replication rate observed might also be the result of inefficient transcellular spreading due to the release of low concentration of seeds over time. Consistent with this, we observed seeding in only 20% of the cellular population in our experiments, while we would expect more cells to show aggregates if transcellular spreading of aggregated species was efficient. This suggests that despite the high number of aggregates present in some cells, these aggregates are not able to reach the extracellular space or enter neighboring cells to induce aggregation. A potential explanation for this is that large fibrillar species might not be able to spread from cell to cell very efficiently, which is further supported by work from previous studies where the reported extracellular tau assemblies are very small in size and mostly monomeric or oligomeric.^13,29,30^ In line with these studies, we report the presence of small tau assemblies in the extracellular space of HEK cells and primary neurons. An alternative explanation is that the process of aggregate replication is largely cell autonomous and slow *in vivo*. Consistent with the latter, we observed that all the unseeded P301S neurons that we imaged would develop small amounts of AT8-positive tau assemblies, the number of which would increase over time. However, we cannot exclude the contribution of additional factors *in vivo* that slow down replication or spreading. Addressing which of these processes drives tau pathology is fundamental to understanding the progression of disease and any mechanism-oriented therapeutic intervention.

In summary, using SR imaging methodologies, we provide clear mechanistic advances in the study of the early stages during seeded tau aggregation inside cells. As key players in the rapid amplification of intracellular tau aggregates, we identify the entry of sufficient numbers of tau assemblies into the cytosol, the subcellular localization of the aggregated tau in close proximity to the microtubules, and the contribution of the proteasome. The templated misfolding of pathological species has been identified as the main determinant in the sequential deposition of aggregated tau during disease progression.^54,55^ Therefore, the initial occurrence of seeds in the cytosol, either due to transcellular spread or by spontaneous misfolding, appears to be the critical step during tau aggregation and remains overall the most promising target for therapeutic interventions in the future.^56^

## Limitations of the study

In all experiments of seeded tau aggregation in this study, heparin-induced recombinant tau fibrils were employed. However, recent studies highlighted that synthetic tau aggregates produced in the presence of heparin do not share the same structural properties as the AD-derived material.^57^ Despite this limitation, the synthetic tau aggregates have been shown to induce tau aggregation in cell-culture systems^15^ and are not detectable by the AT8 antibody, as they are not post-translationally modified. This allows the study of the early stages of tau aggregation without any potential contamination of the exogenously added material in the detected seeding events. Moreover, and in line with previous observations,^58,59^ we observe that the templated seeded aggregation of tau is specific to homotypic interactions and is not induced by other types of aggregates. Thus, our work advances the current knowledge on templated seeded aggregation of the WT and P301S tau protein in intact cells. However, some questions remain unanswered. First, we observe tau seeded aggregation to take place in close proximity to the microtubule cytoskeleton. Unfortunately, we are unable at this stage to determine whether the tau aggregates are associated with the cytoskeleton directly or whether tau aggregates approach the microtubule cytoskeleton to recruit tau monomers in the process of elongation. Furthermore, similar to studies in human iPSC-derived neurons,^51^ we observe faster aggregate amplification in cell-culture models when compared to *in vivo* models.^12,60^ Our cell-culture systems are only viable for a defined amount of time in culture and do not allow the study of tau aggregation in seeded or unseeded cells at later stages, or the spreading of the pathology to neighboring cells, which occurs in longer *in vivo* studies. Overall, we believe that our newly established imaging methodologies combined with similar advances in SR microscopy^61^ will enable the detection of tau aggregates at high resolution in future studies to provide a better understanding of the mechanisms involved at the early stages of tau aggregation.

## Star Methods

### Key Resources Table

**Table:** 

REAGENT or RESOURCE	SOURCE	IDENTIFIER
Antibodies		
Mouse anti-phospho-tau (AT8)	Thermo Fisher Scientific	Cat# MN1020, RRID:AB_223647
Mouse anti-tau (HT7)	Thermo Fisher Scientific	Cat# MN1000, RRID:AB_2314654
Mouse anti-phospho-tau (AT8), biotin	Thermo Fisher Scientific	Cat# MN1020B, RRID:AB_223648
Mouse anti-tau (HT7), biotin	Thermo Fisher Scientific	Cat# MN1000B, RRID:AB_223453
Mouse anti-GAPDH	Thermo Fisher Scientific	Cat# MA5-15738, RRID:AB_10977387
Rabbit Anti-tau polyclonal antiserum KJ9A	Agilent	Cat# A0024, RRID:AB_10013724
Mouse MC1 antibody	Peter Davies Lab	RRID:AB_2314773
Rabbit anti-alpha tubulin	Abcam	Cat# ab18251, RRID:AB_2210057
Mouse anti-alpha tubulin	DHSB	Cat# 12G10, RRID:AB_2315509
Rabbit anti-ubiquitin (linkage-specific K48)	Abcam	Cat# ab140601, RRID:AB_2783797
Goat anti-rabbit Dylight 800	Cell signaling	Cat# 5151, RRID:AB_10697505
Goat anti-mouse Dylight 680	Cell signaling	Cat# 5470, RRID:AB_10696895
Goat anti-Rabbit IgG Alexa Fluor™ 488	Thermo Fisher Scientific	Cat# A-11008, RRID:AB_143165
Goat anti-Rabbit IgG, Alexa Fluor™ 647	Thermo Fisher Scientific	Cat# A-21244, RRID:AB_2535812
Goat anti-Mouse IgG, Alexa Fluor™ 647	Thermo Fisher Scientific	Cat# A-21235, RRID:AB_2535804
Goat anti-Mouse IgG1, Alexa Fluor™ 488	Thermo Fisher Scientific	Cat# A-21121, RRID:AB_2535764
Goat anti-Mouse IgG (whole molecule)–Gold	Merck	Cat# G7652, RRID:AB_259958
Goat anti-Mouse IgG, Alexa Fluor™ 488	Thermo Fisher Scientific	Cat# A-11001, RRID:AB_2534069
Mouse anti-Rabbit	Thermo Fisher Scientific	Cat# 31213, RRID:AB_228376
10 nm Ni-NTA-Nanogold	Nanoprobes	Cat# 2084
Bacterial and virus strains		
AAV1/2-hSyn-eGFP-P2A-LgBiT-NLS	Tuck et al., 2022^20^	N/A
LV-SFFV-human 0N4R tau	This paper	N/A
LV-SFFV-human 0N4R P301S tau	This paper	N/A
BL21(DE3) *E.coli*	Agilent	Cat# 200131
DH10B *E.coli*	Thermo Fisher Scientific	Cat# EC0113
Chemicals, peptides, and recombinant proteins		
Heparin sodium salt	Merck	Cat# H3393; CAS: 9041-08-1
Bafilomycin-A	Merck	Cat# SML1661; CAS: 88899-55-2
Triton X-100	Sigma-Aldrich	Cat# X100
Lipofectamine 3000	Thermo Fisher Scientific	Cat# L3000001
Penicillin-Streptomycin (10,000 U/mL)	Thermo Fisher Scientific	Cat# 15140122
FuGENE 6	Promega	Cat# E2691
MG132	Merck	Cat# M7449
Carfilzomib (CFZ)	Cayman Chemicals	Cat# 17554; CAS: 868540-17-4
Rapamycin	Merck	Cat# 553210; CAS: 53123-88-9
Nocodazole	Merck	Cat# SML1665; CAS: 31430-18-9
Puromycin Dihydrochloride	Thermo Fisher Scientific	Cat# A1113803,
DNAse from bovine pancreas	Merck	Cat# DN25
Cultrex Poly-L-Lysine	R&D Systems	Cat# 3438-200-01
cOmplete™, EDTA-free Protease Inhibitor Cocktail	Merck	Cat# COEDTAF-RO
PhosSTOP™ Phosphatase Inhibitor Cocktail	Merck	Cat# PHOSS-RO
Dimethyl sulfoxide (DMSO)	Sigma-Aldrich	Cat# D8418
RIPA Buffer	Merck	Cat# R0278
NuPAGE™ LDS Sample Buffer (4X)	Thermo Fisher Scientific	Cat# NP0007
2-Mercaptoethanol	Merck	Cat# 805740, CAS: 60-24-2
NuPAGE™ MOPS SDS Running Buffer (20X)	Thermo Fisher Scientific	Cat# NP0001
Bovine Serum Albumin	Thermo Fisher Scientific	Cat# BP9702, CAS: 9048-46-8
Tween-20	Merck	Cat# P1379, CAS: 9005-64-5
6×His-human-0N4R-P301S-tau-HiBiT	This paper	N/A
6×His-human-0N4R-P301S-tau	This paper	N/A
human α-synuclein	This paper	
human amyloid-beta 42 (Aβ42) peptide	rPeptide	Cat# A-1170
Benzamidine hydrochloride hydrate	Merck	Cat# B6506, CAS: 206752-36-5
PMSF	Merck	Cat# PMSF-RO, CAS: 329-98-6
NaCl	Merck	Cat# S9888, CAS: 7647-14-5
HEPES	Merck	Cat# H3375, CAS: 7365-45-9
Imidazole	Merck	Cat# I2399, CAS: 288-32-4
Nonidet™ P40 Substitute	Merck	Cat# 74385, CAS: 9016-45-9
DTT	Merck	Cat# DTT-RO, CAS: 3483-12-3
MgCl2	Merck	Cat# 3483-12-3, 7786-30-3
Ampicillin sodium salt	Formedium	Cat# AMP10
IPTG	Merck	Cat# I6758, CAS: 367-93-1
Tris	Merck	Cat# 93352, CAS: 77-86-1
EDTA	Merck	Cat# 798681, CAS: 60-00-4
DNAse I	Merck	Cat# 10104159001
RNAse	Merck	Cat# 10109169001
HCl	Merck	Cat# 258148, CAS: 7647-01-0
NaOH	Merck	Cat# 567530, CAS: 1310-73-2
NaN_3_	Merck	Cat# 71290, CAS: 26628-22-8
Uranyl acetate	SPI supplies	Cat# 02624-AB, CAS: 6159-44-0
Gelatin from cold water fish skin	Merck	Cat# G7041, CAS: 9000-70-8
Poly-L-Lysine solution	Merck	Cat# P4707, CAS: 25988-63-0
Sodium pyruvate	Thermo Fisher Scientific	Cat# 11360070
GlutaMAX™ Supplement	Thermo Fisher Scientific	Cat# 35050061
Poly-D-Lysine 1.0 mg/ml solution	Merck	Cat# A-003-E
Hoechst 33342, Trihydrochloride, Trihydrate - 10 mg/mL Solution in Water	Thermo Fisher Scientific	Cat# H3570
Paraformaldehyde Solution, 4% in PBS	Thermo Fisher Scientific	Cat# J19943.K2
EGTA	Merck	Cat# 324626, CAS: 67-42-5
Sucrose	Thermo Fisher Scientific	Cat# 10346150, CAS: 57-50-1
N-lauroylsarcosinate sodium salt	Merck	Cat# L9150, CAS: 137-16-6
Skim milk powder	Merck	Cat# 70166
pFTAA	Merck	Cat# SCT066
TetraSpeck™ Microspheres, 0.1 μm	Thermo Fisher Scientific	Cat# T7279
Glucose oxidase	Merck	Cat# 345386, CAS: 9001-37-0
Catalase from human erythrocytes	Merck	Cat# C3556, CAS: 9001-05-2
Cysteamine	Merck	Cat# M9768, CAS: 60-23-1
D-(+)-Glucose	Merck	Cat# G8270, CAS: 50-99-7
β-Galactosidase	Merck	Cat# G3665, CAS: 9031-11-2
β-1,4-galactosyltransferase	Merck	Cat# SAE0093
Glutaraldehyde solution	Merck	Cat# G5882, CAS: 111-30-8
NaBH_4_	Merck	Cat# 213462, CAS: 16940-66-2
Salmon Sperm DNA Solution	Thermo Fisher Scientific	Cat# 15632011
Acetone	Merck	Cat# 179124, CAS: 67-64-1
Methanol	Merck	Cat# 322415, CAS: 67-56-1
KOH	Merck	Cat# 221473, CAS: 1310-58-3
3-Aminopropyltriethoxysilane	Thermo Fisher Scientific	Cat# 10677502, CAS: 919-30-2
Acetic acid	Merck	Cat# A6283, CAS: 64-19-7
NaHCO_3_	Merck	Cat# S6014, CAS: 144-55-8
mPEG-Succinimidyl Valerate, MW 5,000	Laysan Bio Inc.	Cat# MPEG-SVA-5000
Biotin-PEG-SVA, MW 5,000	Laysan Bio Inc.	Cat# Biotin-PEG-SVA-5000
MS(PEG)4 Methyl-PEG-NHS-Ester Reagent	Thermo Fisher Scientific	Cat# 22341
NeutrAvidin Protein	Thermo Fisher Scientific	Cat# 31000
LgBiT protein	Promega	Cat# N2013
Critical commercial assays		
Nano-Glo Live Cell Assay System	Promega	Cat# N2013
PrestoBlue Viability Reagent	Thermo Fisher	Cat# A13261
Human Tau ELISA kit	Abcam	Cat# ab273617
BCA assay	Abcam	Cat# ab287853
CytoTox 96 Non-Radioactive Cytotoxicity Assay	Promega	Cat#G1780
SiteClick™ Antibody Azido Modification Kit	Thermo Fisher Scientific	Cat# S20026
TaqMan Universal PCR Master Mix	Applied Biosystems	Cat# 4305719
Q5 High-Fidelity PCR Kit	New England Biolabs	Cat# E0555L
Quick Ligation Kit	New England Biolabs	Cat# M2200L
PureLink HiPure Plasmid Maxiprep Kit	Thermo Fisher	Cat# K210016
Experimental models: Cell lines		
HEK 293T-NLS-eGFP-LgBiT	Tuck et al., 2022^20^	N/A
HEK 293T-REx-human-0N4R-P301S-tau-venus	McEwan et al., 2017^15^	N/A
HEK 293T-REx-human-0N4R-P301S-tau	This paper	N/A
HEK 293T-REx-human-0N4R-tau	This paper	N/A
HEK 293T-REx	Thermo Fisher	R71007
HEK 293T	ATCC	CRL-3216; RRID: CVCL_0063
Experimental models: Organisms/strains		
C57BL/6 mice	MRC-LMB	RRID: MGI:2159769
Thy1-hTau.P301S mice (CBA.C57BL/6)	MRC-LMB	Allen et al., 2002^26^; RRID: MGI:5450673
Oligonucleotides		
Fw-MIuI-tau: aggatacgcgtgccac catggctgagcc	Merck	N/A
RV_NotI_tau: tagagtgcggccgctta caaaccctgcttggccaggg	Merck	N/A
Docking strand 1 (DS1): DBCO TEG*-TTATACATCTATTTTTTTTTTTTTTTTTTTT	ATDBio	N/A
Docking strand 2 (DS2): DBCO TEG*-TTATCTACATATTTTTTTTTTTTTTTTTTTT	ATDBio	N/A
Docking strand 3 (DS3): DBCO TEG*-TTTCTTCATTA	ATDBio	N/A
Imaging strand 1 (IS1): CTAGAT GTAT-ATTO655	ATDBio	N/A
Imaging strand 2 (IS2): TATGTA GATC-ATTO655	ATDBio	N/A
Imaging strand 3 (IS3): GTAATGAAGA-ATTO655	ATDBio	N/A
Recombinant DNA		
pSMPP-human-0N4R-P301S-tau	This paper	N/A
pSMPP-human-0N4R-tau	This paper	N/A
pAAV-eGFP-P2A-LgBiT-nls	Tuck et al., 2022^20^	N/A
pCRV-Gag-Pol	Prof. Stuart Neil, Kings College London	N/A
pMD2.G	Prof. Didier Trono, EPFL	Cat# 12259; RRID: Addgene_12259
pSMPP	Addgene	Cat# 104970; RRID: Addgene_104970
pRK172-human-0N4R-P301S-tau-HiBiT	Tuck et al., 2022^20^	N/A
pRK172-human-0N4R-P301S-tau	This paper	N/A
pRK172-human-α-synuclein	This paper	N/A
Software and algorithms		
Fiji	Fjii	https://fiji.sc; RRID: SCR_002285
Prism 9	GraphPad	https://www.graphpad.com; RRID: SCR_002798
NIS Elements 4.30	Nikon	https://www.microscope.healthcare.nikon.com/en_EU/products/software/nis-elements; RRID: SCR_014329
Micro-Manager 1.4	Micro-Manager	https://micro-manager.org/; RRID: SCR_000415
ComDet Fiji plugin	Eugene Katrukha	https://doi.org/10.5281/zenodo.4281064;^62^
Python 2.7 scikit-learn 0.18.1	Python	https://pypi.org/project/scikit-learn/0.18.1/
SR toolkit	Whiten et al., 2018^63^	https://github.com/Eric-Kobayashi/SR_toolkit
BioRender	BioRender	https://www.biorender.com/; RRID: SCR_018361
Other		
DMEM High Glucose GlutaMAX Pyruvate	Thermo Fisher Scientific	Cat# 31966047
Fetal bovine serum (FBS)	Thermo Fisher Scientific	Cat# 10270106
Hibernate™-A Medium	Thermo Fisher Scientific	Cat# A1247501
Trypsin (2.5%), no phenol red	Thermo Fisher Scientific	Cat# 15090046
9-inch glass cotton plugged Pasteur pipette	Thermo Fisher Scientific	Cat# 13-678-8B
Neurobasal™ Plus Medium	Thermo Fisher Scientific	Cat# A3582901
Horse Serum	Thermo Fisher Scientific	Cat# 16050122
B-27™ Plus Supplement (50X)	Thermo Fisher Scientific	Cat# A3582801
Nunc™ Lab-Tek™ Chambered Coverglass	Thermo Fisher Scientific	Cat# 155411
NuPAGE™ 4 to 12%, Bis-Tris, 1.0–1.5 mm, Mini Protein Gels	Thermo Fisher Scientific	Cat# NP0324BOX
Trans-Blot Turbo Mini 0.2 μm Nitrocellulose Transfer Packs	Bio-Rad	Cat# 1704158
2xTY	Merck	Cat# Y2377
Carbon Film 400 Mesh grids, Cu, 50/Bx	Electron Microscopy Sciences	Cat# CF400-Cu-50
CO_2_ Independent Medium	Thermo Fisher Scientific	Cat# 18045088
μ-Slide 8 Well	ibidi	Cat# 80826
Nitrocellulose Membrane	Bio-Rad	Cat# 1620112
Bio-Dot® Microfiltration System	Bio-Rad	Cat# 1703938
Amicon Ultra-0.5 Centrifugal Filter Unit	Merck	Cat# UFC5050

## Resource Availability

### Lead contact

Further information and requests for resources and reagents should be directed to and will be fulfilled by the lead contact, David Klenerman (dk10012@cam.ac.uk).

### Materials availability

All unique/stable reagents generated in this study are available from the lead contact with a completed materials transfer agreement.

## Experimental Model And Study Participant Details

### Mice

All animal work was licensed under the UK Animals (Scientific Procedures) Act 1986 and approved by the Medical Research Council Animal Welfare and Ethical Review Body. All animals were housed in pathogen-free conditions with routine veterinary and husbandry procedures carried out. Post-natal day 0 or day 1 wild-type C57BL/6 and Thy1-hTau.P301S (CBA.C57BL/6) male and female pups were used for primary cultures for the tau entry and tau seeding experiments, respectively.

### Cell lines

HEK293T cells were purchased from ATCC (CRL-3216) and HEK293T-REx from Thermo Fisher Scientific (R71007). Both cell lines were maintained in DMEM High Glucose GlutaMAX Pyruvate DMEM supplemented with 10% FBS, 100 U/ml penicillin, 100 ug/ml streptomycin and grown at 37°C and 5% CO_2_.

## Method Details

### Lentivirus-mediated generation of new cell lines

The HEK293 cell lines expressing the P301S tau-Venus and NLS-eGFP-LgBiT were generated previously.^15,20^ The HEK293 cell line expressing untagged WT and P301S tau was generated as described previously.^15^ Briefly, both cDNA constructs were amplified using the primers Fw_MluI_tau: aggatacgcgtgccaccatggctgagcc and RV_NotI_tau: tagagtgcggccgcttacaaaccctgcttggccaggg. The resulting PCR products were cloned into the HIV vector pSMPP (Addgene plasmid #104970) using the indicated restriction enzymes (New England Biolabs). Lentiviral particles were produced using the HIV-1 GagPol expressor pcRV1, the VSV-G glycoprotein expressor pMD2.G (Addgene plasmid #12259) and the Rev expressing plasmid. Plasmids were co-transfected into HEK293 cells, using Fugene-6. After 2 days, supernatant was filtered at 0.45 μm and used to transduce HEK293 cells. Cells expressing untagged tau were selected in the presence of puromycin at 2.5 μg/ml.

### Treatment of cells with inhibitors

The cells were treated with 1 μM MG132 (Merck, M7449) or 1 μM Carfilzomib (Cayman Chemical, 17554) for 16 hours to inhibit the function of the proteasome. Cells were alternatively treated for the same duration with 200 nM Bafilomycin A1 (Merck, SML1661) or 100 nM rapamycin (Merck, 553210) to inhibit or induce autophagy respectively. The disruption of the microtubule network was achieved by treating the cells with 0.25 μM Nocodazole (Merck, SML1665) for 24 hours in combination with the seeding reaction. Assessment of the nocodazole efficiency was performed by immunostaining treated and untreated cells against tubulin (1:500 dilution, DSHB, 12G10).

### Primary neuron culture

Brains were removed from mice and pooled primary neurons were isolated from the cortices and hippocampi as previously described.^20^ Cortices and hippocampi from 6 mice were isolated in cold Hibernate-A medium (Gibco, A1247501) and pooled in a 15 ml conical tube. Tissue was then washed gently twice with 10 ml cold Hibernate-A. Media was removed and replaced with 4.5 ml cold Hibernate-A plus 500 μl 10X Trypsin protease solution (Gibco, 15090046) and incubated at 37°C for 20 min. During incubation, a 9-inch glass cotton plugged Pasteur pipette (Thermo Fisher Scientific, 13-678-8B) was flame polished until the tip resembled the diameter of a P1000 pipette tip. Post-incubation, DNA was digested by the addition of 500 μl 1% DNase (w/v) and incubated at room temperature for 3 minutes. The digested tissue was then washed twice with room temperature Hibernate-A followed by a single wash with pre-warmed plating medium composed of Neurobasal Plus (Gibco, A3582901), 1mM GlutaMAX (Gibco, 35050061), 10% horse serum, 1% penicillin-streptomycin and 1x B-27 Plus (Gibco, A3582801). The tissue was triturated exactly 9 times before straining through a 70 mm cell strainer. Cells were then counted by trypan blue staining. 100,000 viable cells were plated per well into a poly-L-lysine coated (RnD Systems, 3438-100-01) 8-well glass bottom chamber (Labtek, Thermo Fisher Scientific, 155411) in plating medium for 4 h before a complete media change to maintenance medium (plating medium devoid of serum) and maintained in a tissue culture incubator with at 37°C with 5% CO_2_. Media was topped up with 50% of volume on DIV 2.

### Intracellular tau concentration

The concentration of tau in HEK293 cells expressing the untagged P301S and WT tau was determined using the Human Tau ELISA Kit (abcam, ab273617). Shortly, cells were detached from a six well plate and the number of cells as well as their size was quantified using the countess cell counter (Thermo). The cells were subsequently lysed, and ELISA assay was performed according to the manufacturer’s instructions.

To compare the expression levels between the HEK293 cells and the primary neurons, cell lysates were prepared by incubating cells with lysis buffer (PBS buffer containing 1% Triton X-100,1x EDTA-Free Protease Inhibitor mix and phosphatase inhibitor mix) for 30 min on ice. Subsequently the lysate was clarified by centrifugation (14,000 g, 10 min, 4°C). The protein levels were determined using a BCA assay (abcam, ab287853) and 10 ug of protein was loaded on a NuPAGE Bis-Tris 4-12% gels and subjected to SDS PAGE and western blotting. For the detection of the proteins the DAKO antibody (A0024, 1:500) was used for the detection of the tau protein and the GAPDH antibody (Thermo, MA5-15738, 1:5000) was used as a loading control. The proteins were detected using the anti-rabbit Dylight 800 (Cell Signalling, 5151P, 1:5000) and anti-mouse Dylight 680 (cell Signalling, 5470P, 1:5000).

### SDS-PAGE and western blotting

Approximately 100,000 HEK293 cells expressing untagged tau P301S were plated in 24-well plate and the next day the medium was replaced with fresh containing the proteasome inhibitors or DMSO as described above. 16 h later the medium was removed, the cells were harvested by trypsinization, and pelleted by centrifuging at 500 g for 5 min at room temperature. The cells were lysed on ice for 15 min in 80 μl 1X RIPA buffer (Merck, R0278) with 1x protease (Merck, 11873580001) and 1x phosphatase (Merck, 4906845001) inhibitors. The lysates were centrifuged (14,000 g, 15 min, at 4 °C) and 75 μl of clarified lysate was mixed with 25 μl 4× NuPAGE LDS sample buffer (Thermo Fisher Scientific, NP0007) containing 2 mM b-mercaptoethanol. The final samples were boiled at 100 °C for 5 min and subjected to SDS-PAGE using NuPAGE Bis-Tris 4-12% gels (Thermo Fisher Scientific, NP0324BOX) in MOPS-SDS running buffer for 55 min at 200 V. The gel was electroblotted onto a 0.2 μm nitrocellulose membrane (Bio-Rad, 1704158) using the Bio-Rad Transblot Turbo Transfer System. The transferred membranes were blocked in 3% BSA (Fisher BioReagents, BP9702) diluted in 0.2% Tween-20 PBS (PBST) for 1 h at room temperature and then incubated with the primary antibody overnight at 4°C. After repeated washes with PBST, the membranes were incubated with secondary Alexa-conjugated secondary antibodies (Thermo Fisher Scientific, A-11008, A-21244, A-21235, and A-21121) at 1:2000 dilution for 1 h at room temperature. Finally, the membranes were imaged using a ChemiDoc gel imager equipment and densitometrically quantified using Fiji/ImageJ.^64^

### Preparation of recombinant assemblies

Recombinant N-terminally 6xHis-tagged human P301S 0N4R tau and 6xHis-tagged human P301S-0N4R-tau-HiBiT were expressed and purified from *E. Coli* BL-21 DE3 cells. Protein expression was performed at 16°C overnight. Cells were pelleted (17,000 × g, 3 min) and lysed in tau-lysis buffer (1 mM benzamidine, 1 mM PMSF, 1× EDTA-Free Protease inhibitors mix (Merck), 14 mM b-mercaptoethanol, 300 mM NaCl, 25 mM HEPES, 30 mM imidazole, 1% NP-40). Purification was performed on the AKTA Pure using the HisTrap HP column (Cytiva), followed by size exclusion chromatography using a Superdex 200 HiLoad 16/600 pg column as previously described.^13^ All the proteins were stored in PBS Buffer freshly supplemented with 1 mM DTT. Assemblies were prepared by addition of heparin at 37°C for 3 days while shaking, using tau at 60 μM in the presence of 20 μM heparin (Sigma Aldrich) in PBS supplemented with 2mM DTT and 1× EDTA-Free Protease Inhibitor mix. A small aliquot of the assemblies was kept for analysis and the remaining material was sonicated for 15 sec before long-term storage at –80°C.

Recombinant α-synuclein was expressed and purified as described previously.^65^ Briefly, full-length human α-synuclein was expressed in *E*. *coli* BL21 (DE3) (Agilent Technologies, 200131) using the plasmid pRK172. The bacteria were grown in 2xTY medium containing 5mM MgCl2 and 100 mg/L ampicillin at 37°C until an OD600 of 0.7 was reached. Then, α-synuclein expression was induced with 1 mM IPTG and after 4 hrs at 37°C the cells were harvested by centrifugation. The pellets were resuspended in cold α-synuclein-lysis buffer(50 mM Tris-HCl, pH 7.5,10 mM EDTA, 40 μg/ml DNase (Merck, 10104159001) and 10 μg/ml RNase (Merck, 10109169001), supplemented with cOmplete EDTA-free Protease Inhibitor Cocktail (Merck, 11873580001)). Subsequently, they were sonicated on ice using a Sonics VCX-750 Vibra Cell Ultra Sonic Processor for 5 min (5 s on, 10 s off) at 90% amplitude. The lysates were centrifuged at 20,000 g for 40 min at 4 °C and filtered with a 0.45 μm cut-off filter. The pellets were discarded and the pH of the supernatant was lowered to 3.5 with HCl, stirred for 30 minutes at RT and centrifuged at 50, 000 g at 4°C for 1 hr. Subsequently, the pH was increased to 7.5 by adding NaOH to the supernatant. Supernatants were loaded onto an anion exchange HiTrap Q HP column and eluted with a 0-1 M NaCl gradient, which was followed by size exclusion chromatography using a Superdex 200 HiLoad 16/600 pg column. The purity of α-synuclein was analyzed by SDS-PAGE and the protein concentration was determined spectrophotometrically using an extinction coefficient of 5600 M^–1^ cm^–1^. The purified monomer was stored in PBS and assembled into filaments by shaking at 200 rpm for 5 days at 37°C at a concentration of 357 μM. The assemblies were sonicated for 15 sec and stored at -80°C.

Lyophilized monomeric recombinant Aβ42 peptide (Stratech, Cat. No. A-1170-2-RPE-1.0mg) was dissolved in PBS (pH = 7.4) at 200 μM on ice. The solution was quickly aliquoted and snap frozen. To prepare recombinant Aβ42 fibrils, an aliquot was thawed and diluted to 4 μM in 1× PBS supplemented with 0.01% NaN_3_ (Merck, Cat. No. 71290) and incubated at 37 °C under quiescent conditions for one week. The Aβ42 fibril was then sonicated as described previously^66^ with modifications. The one-week aggregated Aβ42 aliquot was immersion sonicated in an ice water bath with a 3-mm-titanium probe (Sonicator microprobe 4422, Qsonica) mounted on a tip sonicator (Ultrasonic processor Q125, QSonica) at 20 kHz with 40% of power for 24×5-s bursts with 15-s rests between bursts. Thereafter, the sonicated aggregate was centrifuged, aliquoted (50 μl) and snap frozen. The aliquots were stored at –80°C until use.

### Immunogold labeling and negative-stain electron microscopy of recombinant and cell-derived fibrils

Heparin-assembled recombinant fibrils before and after sonication at a concentration of 1μM as well as Sarkosyl-resistant species from seeded cells were applied on glow-discharged 400 mesh formvar/carbon film-coated copper grids (EM Sciences, CF400-Cu) for 45 sec. For negative stain analysis, the excess liquid was removed, the grids were stained with 2% uranyl acetate for 45 sec, and air-dried for 30 min before image acquisition. The immunogold labeling was performed as described previously.^67^ Briefly, the grids with deposited samples were blocked at room temperature for 10 min with PBS + 0.1% cold fish skin gelatin (G7041, Merck) and then incubated with the AT8 primary antibody (1:50) diluted in blocking buffer for 1 h at room temperature. The grids were subsequently washed with blocking buffer and incubated with 10 nm gold-conjugated anti-mouse IgG secondary antibody (G7652, Merck) for 1 h at room temperature diluted 1:20 in blocking buffer. For detection of 6xHis-tag epitopes, 10 nm Ni-NTA-Nanogold particles (Nanoprobes, 2084-3ML) were employed for labeling for 1 h at room temperature after dilution in blocking buffer (1:25). The grids were finally washed with water, stained with 2% uranyl-acetate for 45 sec and air-dried for at least 30 min before imaging. Images were acquired at 4,400× and 6,500× with a defocus value of -1.4 μm with Gatan Orius SC200B detector using a Tecnai G2 Spirit at 120 kV. Fibril widths were measured manually using the Fiji software for at least 100 fibrils.

### Tau entry

The cytosolic entry of tau in HEK293 and primary neurons was quantified using the previously established assay as described in.^20^ For HEK cells, 2 × 10^4^ cells expressing NLS-eGFP-LgBiT were seeded into white 96-well plates (Greiner bio-one, 655098) coated with poly-L-lysine (Sigma, P4707) in complete DMEM. 12–16 h later, the medium was replaced with 50 μL serum free CO_2_ independent medium (Thermo Fisher, 18045088) supplemented with 1 mM sodium pyruvate, 1% penicillin-streptomycin, 1 mM GlutaMAX and recombinant tau-HiBiT fibrils, added in 100 μL. After incubation with tau in the presence of lipofectaminefor 24 hours, the media was aspirated, and cells washed once with PBS. PBS was aspirated and replaced with CO_2_ independent medium plus live cell substrate according to manufacturer instructions (Promega, N2013). The cells were incubated for 5 min and immediately loaded onto the ClarioSTAR plate reader where luminescent signal was quantified at 37 °C.

To quantify the entry of tau in primary neurons, 30,000 primary neurons from postnatal day 0/1 C57BL/6 mouse pups were seeded per well into a white poly-L-lysine coated 96-well plate. The neurons were infected at DIV 2 with AAV1/2 hSyn::-eGFP-P2A-LgBiT-NLS particles at a multiplicity of 50,000 genome copies per cell. On DIV 7, neurons were subjected to a full media change with fresh maintenance medium supplemented with 100 nM of tau-HiBiT. Signal quantification was performed as described for the HEK293 tau entry assay above.

The entry of tau was calculated as % of the amount of added tau derived from incubation of 100 μl of 100 nM recombinant tau aggregates incubated with excess of recombinant LgBiT in a separate well.

To compare the entry of tau in the presence or absence of proteasome inhibitors, HEK293 cells expressing the NLS-eGFP-LgBiT were treated with 100 nM recombinant tau-HiBiT fibrils for 4 hours in the presence of proteasome inhibitors or while mock treated.

### Seeding assays

For super-resolution experiments, HEK293 cells expressing WT tau, P301S tau or P301S tau-Venus were plated in 8-well glass bottom chambers (ibidi) pre-treated with 0.1% poly-L-lysine (Sigma Aldrich) and allowed to adhere overnight in full medium. The next day the medium of each well was exchanged with 200 μl full medium containing the indicated amount of recombinantly produced tau assemblies and 1 μl of each component of the Lipofectamine 3000 reagent (Thermo Fisher Scientific) unless indicated otherwise. The cells were incubated for the indicated time and subsequently fixed using ice-cold methanol for 3 min at room temperature.

Primary neurons were supplemented with a final concentration of 100 nM tau assemblies at DIV 7 in maintenance medium and incubated at 37°C and 5% CO_2_. The cells were incubated for the indicated time and subsequently fixed using ice-cold methanol for 3 min at room temperature. To determine the proportion of tau assemblies detected in the soma of the neurons, the total number of assemblies detected in the neuronal soma as well as in the whole FOV were quantified using the ComDet plugin^62^ in Fiji.

Second generation and nocodazole-seeding experiments were performed as described previously with small modifications.^15^ Approximately 20,000 cells were plated in black 96-well plates pre-coated with poly-D-lysine (Merck, A-003-E, final coating concentration of 50 μg/ml) and left to adhere overnight. The next day, the cells were rinsed with PBS and were added 100 μl fresh medium containing the indicated amounts of recombinant or cell-extracted assemblies in complex with 0.5 μl of each component of the Lipofectamine 3000 reagent (Thermo Fisher Scientific, L3000015). The cells were incubated at 37 °C for 24 h after the addition of fibrils and then were fixed with ice-cold methanol for 3 min at room temperature. The nuclei were stained with 1 μg/ml Hoechst 33342 (Thermo Fisher Scientific, H3570) for 10 min and images were acquired at 405 and 488 nm on a Ti2-E High Content Microscope (Nikon). Nine fields per well were read in a horizontal serpentine acquisition mode with a 10× objective and the downstream analysis was performed using the Fiji software^64^. For nuclear counting, the 405 nm acquired images were locally subtracted for background using the Rolling ball algorithm subtraction and the cells were segmented based on nuclear staining using the Median filter and Find Maxima tools, with Segmented Particles above lower threshold option activated. The seeded aggregates at the 488 nm images were detected and quantified using the ComDet plugin^62^ in Fiji. The relative levels of seeding were calculated as the number of detected puncta/aggregates in each field normalized to the corresponding number of cells and then compared to the untreated control.

To determine the percentage of seeded cells, HEK293 P301S tau-Venus cells were plated at 10,000 cells per well in black 96-well plates pretreated with 0.1% poly-L-lysine (Sigma Aldrich) and allowed to adhere overnight. The next day the medium of each well was exchanged with 100 μl fresh medium containing 100 nM recombinantly produced tau assemblies and 1 μl of each component of the Lipofectamine 3000 reagent (Thermo Fisher Scientific, L3000015). Cells were incubated at 37°C for another 24 h and subsequently fixed using 4% paraformaldehyde (Thermo Fisher Scientific) and stained with Hoechst 33342 at 1 μg/ml in PBS. Images were taken using a 10× objective lens on a Nikon Ti-E inverted fluorescence microscope, using automated x,y positioning and autofocus, as described previously.^15^ Cells were selected using thickened regions of interest surrounding nuclei, and tau-Venus aggregates were identified using local contrast filters. Threshold levels for detection of aggregates were adjusted using control cells for each experiment. Levels of seeding were calculated as (cells containing aggregates)/(total cells) × 100 for individual fields. All analysis was performed using the NIS Elements 4.30 (Nikon) software.

### Sarkosyl-extraction of insoluble tau species

Sarkosyl-insoluble tau from cells was extracted as described previously^67^ with small modifications. Approximately 600,000 empty or tau P301S-Venus expressing cells were plated in 6-well plates and left to adhere overnight. The next day, the cells were treated with recombinant tau P301S seeds at a final concentration of 100 nM in complex with 7.5 μl of each Lipofectamine3000 component (Thermo Fisher Scientific, L3000015) and altogether diluted in 1.5 ml DMEM fully supplemented with FBS and antibiotics. An equivalent PBS-mock control was included for the untreated condition. The seeding reaction was left for 24 h and then the cells were harvested by trypsinization in order to remove the majority of the non-internalized extracellular recombinant assemblies.^17^ Cells from 3 wells were combined and pelleted by centrifugation (500 g, 5 min, room temperature). Each pellet was homogenized by vigorous pipetting in 3 ml (1 ml/well) of H-buffer (10 mM Tris HCI (pH 7.4), 0.8 M NaCI, 1 mM EGTA, 10% sucrose, and 1% N-lauroylsarcosinate) and sonicated with a Microson XI-2000 Ultrasonic Cell Disruptor (Misonix) for 30 sec. The lysates were incubated for 30 min at 37°C while shaking and then clarified by centrifugation (10,000 g, 10 min, 4 °C). The clarified lysates were then subjected to ultracentrifugation for 1 h at 100,000 g at 4 °C and the supernatants were retained as the “soluble” fraction. The pellet was washed with PBS and re-centrifuged under the same ultracentrifuge conditions. This supernatant from this washing step was discarded and the pellet (“insoluble” fraction) was resuspended in 20 μl resuspension buffer (50 mM Tris HCl, 150 mM NaCl). For second generation seeding, 0.1μl from each condition was employed.

### Cytotoxicity analysis

The potential cytotoxic effects of the treatment of HEK293 cells with tau aggregates were determined by measuring the LDH activity in the conditioned medium, using the CytoTox 96 Non-Radioactive Cytotoxicity Assay (Promega, G1780) according to the manufacturer’s instructions. The cell death levels were calculated as % of the maximum LDH activity derived from the lysed cells.

### Dot-blot analysis

Cell lysates were prepared by incubating cells derived from a 6-well plate with 100 μl of lysis buffer (PBS buffer containing 1% Triton X-100, 1× EDTA-Free Protease Inhibitor mix and phosphatase inhibitor mix) for 30 min on ice. The lysate was clarified by centrifugation (14,000 g, 10 min, 4 °C) and applied to a 0.2 μm nitrocellulose membrane (Bio-Rad, 1620112) using the Bio-Dot microfiltration apparatus (Bio-Rad, 1703938) according to the manufacturer’s instructions. The membranes were then blocked in 5% milk in TBST and then incubated overnight with the KJ9A pan-tau (Agilent, A0024) and the phospho-specific AT8 (Thermo Fisher Scientifics, MN1020) antibodies. The next day the membranes were washed three times with TBST and incubated for 1 h at room temperature with goat anti-mouse Alexa488- (Thermo Fisher Scientific, A11001) and goat anti-rabbit Alexa647-conjugated (Thermo Fisher Scientific, A21244) secondary antibodies for detecting phospho- and pan-tau species, respectively. At the end of the incubation, the membranes were rinsed again three times with TBST and finally imaged using the ChemiDoc system (Bio-Rad).

### Diffraction-limited immunofluorescence imaging of cells

HEK293 or primary neuronal cells were fixed using ice-cold methanol for 3 min at RT. Then, the cells were rinsed three times with PBS and blocked for 1 h with 1% gelatin (Merck, G7041-100G) diluted in PBS (blocking buffer). For antibody staining, the fixed cells were incubated with the primary antibodies overnight at 4 °C and the next day they were washed three times with PBS before staining with the secondary Alexa Fluor-conjugated antibodies for 1 h at RT. Staining of cells with the amyloid-specific dye pFTAA^27^ was performed by incubating the fixed cells for 30 min at RT in a final concentration of 33 nM diluted in PBS. The nuclei were stained with 1 μg/ml Hoechst 33342 (Thermo Fisher Scientific, H3570) for 10 min at RT. Images were acquired at 405, 488, and 647 nm on a Ti2 inverted fluorescence microscope (Nikon) using a 10× and 63× objective.

### Super-resolution imaging of cells

Cells were fixed using ice-cold methanol for 5 min on ice. The cells were subsequently rinsed three times with PBS, blocked for 1 h with 1% Gelatin (Merck, G7041-100G) in PBS (blocking buffer) and incubated overnight with the AT8 antibody (Thermo Fisher Scientific, MN1020) except for SF1 for which the MC1 antibody was employed. Both primary antibodies were used in a dilution of 1:500 in blocking buffer. The next day the cells were rinsed three times with PBS and subsequently incubated with secondary antibodies coupled to Alexa Fluor 647 (Thermo Fisher Scientific, A32728) diluted in blocking buffer for 30 min in room temperature at a concentration of 4 ug/ml. Tetraspeck beads (Thermo Fisher Scientific, T7279, 1:2,000) were added during the incubation with the secondary antibody and subsequently used as fiducial markers for drift correction.

Imaging was performed using highly inclined and laminated optical sheet (HILO) illumination. The imaging solution for dSTORM was prepared as previously reported,^68^ containing 0.5 mg/ml glucose oxidase, 40 μg/ml catalase, 50 mM cysteamine, and 10% glucose in 50 mM Tris supplemented with 10 mM NaCl at pH 8. This solution was prepared freshly and immediately before imaging. A minimum of 16,000 frames with an exposure time of 33 ms was recorded. Every field of view (FOV) (54.8 × 54.8 μm) was selected in order to include one seeded cell that was imaged every time.

### Antibody labeling for DNA-PAINT imaging

Lyophilized DBCO (DBCO TEG – dibenzocyclooctyne tetraethylene glycol) modified docking strands (DS) received from the supplier (ATDBio, Southampton, UK) were dissolved in water to give ~1 mM stock solutions as confirmed by A_260_. The F_c_ part of the antibody was covalently coupled to the docking strand via copper-free click chemistry with the aid of SiteClick Antibody Azido Modification Kit (Thermo Fisher Scientific, S20026). Briefly, 250 μg of the antibody was initially concentrated, then buffer-exchanged to ~2 mg/ml and subsequently treated with β-galactosidase to modify carbohydrate domains overnight. Subsequently, azide modified sugars were attached to the modified glycan chains through an overnight incubation, in the presence of β-1,4-galactosyltransferase. The next day, the antibody was purified using a 50 kDa Amicon Ultra spin column (Merck). The DBCO-modified ssDNA docking strands were mixed at a 10:1 molar ratio with the antibody and incubated overnight at 37 °C. The final product was purified using a 50 kDa Amicon Ultra spin column. The final concentration of the antibody was quantified by A280. The degree of labeling for AT8 and mouse anti-rabbit (labeled with DS1 and DS2, respectively) were quantified by reducing SDS-PAGE (2.32 and 3.2 docking strands per antibody, respectively), while that for HT7 (labeled with DS3) was determined by A_260_/A_280_ (3.6 docking strands per antibody).

### Preparation of imager strands

Imager strands (IS) were purchased from ATDBio (Southampton, UK). They were synthesized on the 1.0 μmol scale and purified by double high-performance liquid chromatography. Lyophilized oligonucleotides were dissolved in 18.2 MU cm water (filtered by 0.02 μm filter [VWR, 516-1501]) to concentrations of 50–1,000 μM as confirmed by A_260_, aliquoted, and stored at -20 °C. They were used at a final concentration of 1-10 nM.

### Exchange DNA-PAINT

HEK293 cells were plated in eight-well glass bottom chambers (ibidi) pre-treated with 0.1% poly-L-lysine (Merck) and 24 hours later they were treated with 100 nM tau fibrils in the presence of 0.5% lipofectamine 3000. The next day the cells were initially incubated with an extraction solution (0.25% Triton, 0.1% glutaraldehyde in PEM, pre-heated to 37°C) for 15–45 s and subsequently fixed for 10 min using the fixation solution (0.25% Triton, 0.5% glutaraldehyde in PEM, preheated to 37°C). For both steps, electron-microscopy grade glutaraldehyde was used (Merck, ref. G5882). The samples were then washed 3 times with PBS and incubated with 0.1% NaBH_4_ in PBS for 7 min at room temperature. This was followed by a 30 min blocking step using blocking buffer containing 0.5% fish gelatin as well as 1 mg/ml Salmon Sperm DNA. The cells were immunostained overnight using an anti-tubulin rabbit anti-body (ab18251) diluted 1:300 and the tau antibody AT8-DS1 0.1 ug/ml in blocking buffer. The next day the cells were rinsed three times with PBS and subsequently incubated with a mouse anti-rabbit antibody (Thermo Fisher Scientific, 31213) labeled with DS2 2 ug/ml for 30 min in room temperature in the presence of Tetraspeck beads diluted in 1:2,000 (Thermo Fisher Scientific, T7279). Imaging was performed sequentially for the tau aggregates and the microtubules, using IS1 (CTAGATGTAT-ATTO655) or IS2 (TATGTAGATC- ATTO655) respectively in PBS supplemented with 500 mM NaCl. An exposure time of 50 ms was used and 16,000 frames were recorded.

### Preparation of SiMPull slides

Glass coverslips (24 × 50 mm, thickness 0.13–0.17 mm, VWR) were covalently PEGylated according to previous studies,^69,70^ with some modifications. Briefly, coverslips were washed by sonication (Ultrasonic cleaner USC100T, VWR) in a series of solvents (10 min in each of 18.2 MΩ/cm water, acetone, then MeOH, followed by 20 min. in 1 M KOH), rinsed with 18.2 MΩ/cm water, then MeOH, then dried in a stream of nitrogen and finally cleaned with argon plasma for 15 minutes (Femto Plasma Cleaner; Diener Electronic, Royal Oak, MI, USA). The surfaces were subsequently silanized with a 1.5:2.5:50 ratio of 3-aminopropyl triethoxysilane (Thermo Fisher Scientific, 10677502), acetic acid, and MeOH, respectively; coverslips were immersed in this solution for 22 minutes, with 1 minute of sonication at the first and 11th minute. The coverslips were then rinsed twice with 18.2 MΩ/cm water, then MeOH, then dried in a stream of nitrogen and then attached to a 50-well PDMS chamber (CultureWell™ Chambered Coverglass, Sigma, GBL103350-20EA). Each well was passivated by adding 10 μl of an aqueous mixture containing 0.1 M NaHCO3 (pH 8.5) and a 99:1 ratio of methoxy- (110 mg/ml, ~22 mM, MW ~5,000, Laysan Bio Inc., AL, USA, MPEG-SVA-5000) and biotin-terminated (1.1 mg/ml, ~220 μM, MW ~5,000, Laysan Bio Inc., AL, USA, Biotin-PEG-SVA-5000) PEG, each activated at the other terminus as the N-hydroxysuccinimidyl ester. After overnight incubation in a humid chamber, the coverslips were rinsed well with 18.2 MΩ/cm water and dried with a stream of nitrogen. Each well was further passivated by adding 10 μl of an aqueous mixture containing 0.1 M NaHCO_3_ (pH 8.5) and a shorter, methoxy-terminated PEG, activated at the other terminus as the N-hydroxysuccinimidyl ester (10 mg/ml, 30 mM, MS(PEG)_4_ Methyl-PEG-NHS-Ester, ThermoFisher, 22341). The coverslips were again incubated overnight in a humid chamber, rinsed well with 18.2 MΩ/cm water, dried with a stream of nitrogen and stored in a desiccator at -20 °C until use.

### SiMPull assay

Assay wells were coated with 10 μl of 0.2 mg/ml NeutrAvidin (Thermo Fisher Scientific, 31000) in TBS containing 0.05% tween-20 (TBST) for 10 min, washed two times with 10 ul TBST and incubated with 10 μl TBS containing 1% tween for 5 min. The wells were subsequently treated with biotinylated AT8 antibody in a concentration of 10 nM in blocking solution (TBST containing 10 mg/ml BSA (Fisher BioReagents, BP9702), followed by two washing steps with TBST, a 5 min incubation with 10 μl TBS containing 1% tween-20 and a final blocking incubation with blocking solution for 10 min. The corresponding cell lysate or media sample was subsequently added in each well and the sample was incubated for 1 h or overnight. Two washing steps with TBST followed, as well as a 5 min incubation with TBS containing 1% tween-20. The wells were finally incubated with 5 nM AT8 antibody labeled with DS1 for media samples and 2 nM HT7 antibody labeled with DS3 for cell lysates, both of which were in blocking solution, followed by two washing steps with TBST and a 5 min incubation with TBS containing 1% tween-20. The buffer was finally exchanged with 5 μl TBS containing 2 nM IS1 or IS3 (GTAATGAAGA-ATTO655). To prevent evaporation the multiwell chamber was sealed on top with another clean coverslip. The slides were then transferred to the microscope stage for imaging. The open-source software Micro-Manager 1.4 was used to automate image acquisition. Images were acquired for 6000 frames in an unbiased way at maximum laser power with an exposure time of 100 ms each.

Cell lysates were prepared by incubating cells derived from a 6-well plate with 100 ul of lysis buffer (PBS buffer containing 1% Triton X-100, 1× EDTA-Free Protease Inhibitor mix and phosphatase inhibitor mix) for 30 min on ice. The lysate was clarified by centrifugation (14,000 g, 10 min, 4°C) and subsequently stored at –80°C, while the cell media was clarified by centrifugation (6,000 g, 10 min, 4°C) and stored at –80°C.

To characterize and determine the number of recombinant tau aggregates administered on the cells, 10 ul of aggregates in a concentration of 100 nM were pulled down on a SiMPull slide. A SiMPull assay was performed as described here using 10 nM biotinylated HT7 antibody for capturing the recombinantly produced seeds on the glass surface and 2 nM HT7 antibody labeled with DS3 in combination with 2 nM of IS3 for the detection of the aggregates by DNA-PAINT. The number of aggregates contained on the glass surface (7 mm^2^ surface area) was calculated. The results were used to determine the number of recombinant aggregates added in the cell supernatant (200 μl) and the number of aggregates per cell was determined.

### Instrumentation

A home-built total internal reflection fluorescence (TIRF) microscope was used for imaging. Four lasers operating at 405 (Cobolt 06-MLD-405, HÜBNER), 488 (Cobolt 06-MLD-488, HÜBNER), 561 (Cobolt 06-DPL-561, HÜBNER), and 638 nm (Cobolt 06-MLD-638, HÜBNER) were coupled to the optical axis of a 1.49 N.A., 60× TIRF objective (Apo TIRF, Nikon) mounted on an inverted Ti-2 Eclipse microscope (Nikon, Japan). The laser power was controlled by their corresponding software or attenuated by neutral density filters. The laser beams then passed through a quarter-wave plate for circular polarization and were cleaned up by their corresponding excitation filter (for 405 nm: FF01-417/60-25x36, Semrock; for 488 nm: LL01-488-25x36, Semrock; for 561 nm: FF01-561/14-25x36, Semrock; for 638 nm: FF01-640/14-25x36, Semrock). The laser beams were then expanded and collimated by beam expanders before reaching their corresponding dichroic mirror (for 405 nm: FF458-Di02-25x36, Semrock; for 488 nm: FF509-FDi01-25x36, Semrock; for 561 nm: FF605-Di02-25x36, Semrock). Next, the reflected and combined laser beams were focused by an achromatic doublet lens (AC254-400-A, Thorlabs) and reflected by a quad-band dichroic beam splitter (Di01-R405/488/561/635-25x36, Semrock). The objective then focused the reflected excitation beam on the sample. Fluorescence was collected by the objective and passed through the quad-band dichroic beam splitter. It was then cleaned up by corresponding appropriate emission filter (for both 405- and 488-nm-induced fluorescence: BLP01-488R-25x36, Semrock and FF01-520/44-25x36, Semrock; for 561-nm-induced fluorescence: LP02-568RS-25x36, Semrock and FF01-587/35-25x36, Semrock; for 638-nm-induced fluorescence BLP01-635R-25x36, Semrock) mounted on motorized BA filter wheel for stage up (Ti2-P-FWBS-E, Nikon) before being recorded on an EMCCD camera (Evolve 512, Photometrics) operating in frame-transfer mode (electron-multiplying gain of 6.3 electrons/ADU and 250 ADU/photon). Each pixel corresponds to a length of 107 nm on the recorded image. The microscope was also fitted with a perfect focus system that auto-corrects the z-stage drift during a prolonged period of imaging.

### Data analysis

The positions of the fluorescent signal within each frame were determined using the PeakFit plugin (an ImageJ/Fiji plugin of the GDSC Single-Molecule Light Microscopy package (http://www.sussex.ac.uk/gdsc/intranet/microscopy/imagej/gdsc_plugins) for ImageJ^64^ using a typical ‘signal strength’ threshold of 150 and a precision threshold of 30 nm. The localizations were sorted into clusters using the DBSCAN algorithm in Python 2.7 (sklearn v0.18.1) using epsilon = 3 pixels and a minimum points threshold of 10 to remove spurious localizations. For each localization in the cluster, the shortest distance to the neighboring localizations was calculated and defined as the nearest neighbor (NN) distance. Molecular positions were plotted and color-coded on basis of the local density, defined as the number of molecules within a radius of 5 times the mean nearest neighbor distance of all molecules in that cluster. The number of clusters, localizations per cluster and nearest neighbor analyses were determined using custom scripts for Igor Pro (Wavemetrics). The resolution was determined by plotting a Fourier ring correlation curve (GDSC SMLM package)^71^ for each image and determining the spatial frequency at which the curve drops below 1/7.^72^ The lengths of the aggregates were calculated to structurally characterize the super-resolved aggregates. Using a custom script in Python 2.7 the clusters were skeletonized using SciPy v0.18.1 and their length was calculated as reported previously.^63^ The code that was used for super-resolution analysis is open-source and publicly available on https://github.com/Eric-Kobayashi/SR_toolkit as previously described in Whiten et al., 2018. The length and the eccentricity values of each cluster were automatically generated following clustering. The eccentricity of a cluster is calculated by fitting an ellipse to the cluster and determining the focal distance of the ellipse divided by the maximum distance of the major axis. All clusters having an eccentricity value > 0.9 were classified as fibril-like structures based on previous observations.^25,73^

### Kinetic analysis

The mean numbers of aggregates per field of view were fitted with a sigmoidal function.

$$\frac{a}{1+exp\left(-\kappa \left(t-t_{0}\right)\right)}$$

which is linked to the underlying molecular mechanism as outlined previously.^74,75^ This strategy allows extraction of the initial replication rate, κ, also when the aggregate concentrations plateau at later times. The constants *a* and *t*_0_ account for the plateau level and initial seed concentration, respectively. The doubling times are then computed as

$$t(doubling)=\frac{\ln(2)}{k}$$

In order to estimate the confidence in the determined doubling times, given the data, we performed Bayesian inference on the different datasets. We assumed measurements were normally distributed, with mean given by the above function and the standard deviation determined from the data at each time point (i.e. noise is not homoscedastic between different timepoints). We then inferred κ, *a* and *t_0_*, using flat priors for κ, *a* and log(*t*_0_). The maximum likelihoods and 95% confidence intervals for κ are given in Table S1.

In Manos et al.^51^ the aggregate concentrations are determined at both 4 weeks and 7 weeks, for different initial seed concentrations. From these 2 measurements, assuming exponential increase in the interim, one can estimate the replication rate in units of days –1 via:

$$k=\frac{\ln(A7)-\ln(A4)}{21}$$

where A7 and A4 are the concentrations of aggregates at 7 weeks and 4 weeks, respectively. This calculation was performed for the data in Figure 4D, as shown in Table S2. There is some variation for different seed values, so we estimate the doubling time in this system as approximately 2 weeks. Note that our calculations in the main text determine the rate of doubling of the aggregate number, whereas these data are on the aggregate mass instead. As the average size of aggregates appears to increase over the course of the reaction, the doubling time for the aggregate number in this system is likely to be marginally higher than the 2 weeks estimated for the doubling of mass.

### Graphical methods

The graphical abstract was generated using Biorender.

## Quantification and Statistical Analysis

All statistical analyses were performed via GraphPad Prism software. Differences between multiple means were tested by one-way ANOVA, followed by Tukey’s post hoc test unless otherwise indicated in the Figure legend. Differences between two means were tested by unpaired Student’s t test with Welch’s correction. All data represent mean values ± the standard deviation (SD) with the following significances ns p > 0.05, *p < 0.05, **p < 0.01, ***p < 0.001, ****p < 0.0001.

## Supplementary Material

Figures S1-S7 & Tables S1 and S2

Video S1

## Data Availability

All data reported in this paper will be shared by the lead contact upon request. This paper reports no original code. Any additional information required to reanalyze the data reported in this paper is available from the lead contact upon request.

## Highlights

- Treatment with tau fibrils induces the formation of intracellular tau aggregates
- Tau seeded aggregation takes place in the vicinity of the microtubule cytoskeleton
- Spontaneous tau aggregation occurs in cells
- The proteasome accelerates the tau seeded aggregation
