# Supplementary material for: Super-resolution imaging unveils the self-replication of tau aggregates upon seeding
Source: Cell Rep. Author manuscript; Available in PMC 2023 Aug 10. (PMC7614924; doi:10.1016/j.celrep.2023.112725)
Supplement: Figures S1-S7 & Tables S1 and S2 [file EMS184637-supplement-Figures_S1_S7___Tables_S1_and_S2.pdf]

Cell Reports, Volume *42*

Supplemental information

Super-resolution imaging unveils the self-replication of tau aggregates upon seeding

Eleni Dimou, Taxiarchis Katsinelos, Georg Meisl, Benjamin J. Tuck, Sophie Keeling, Annabel E. Smith, Eric Hidari, Jeff Y.L. Lam, Melanie Burke, Sofia Lövestam, Rohan T. Ranasinghe, William A. McEwan, and David Klenerman

Supplementary information

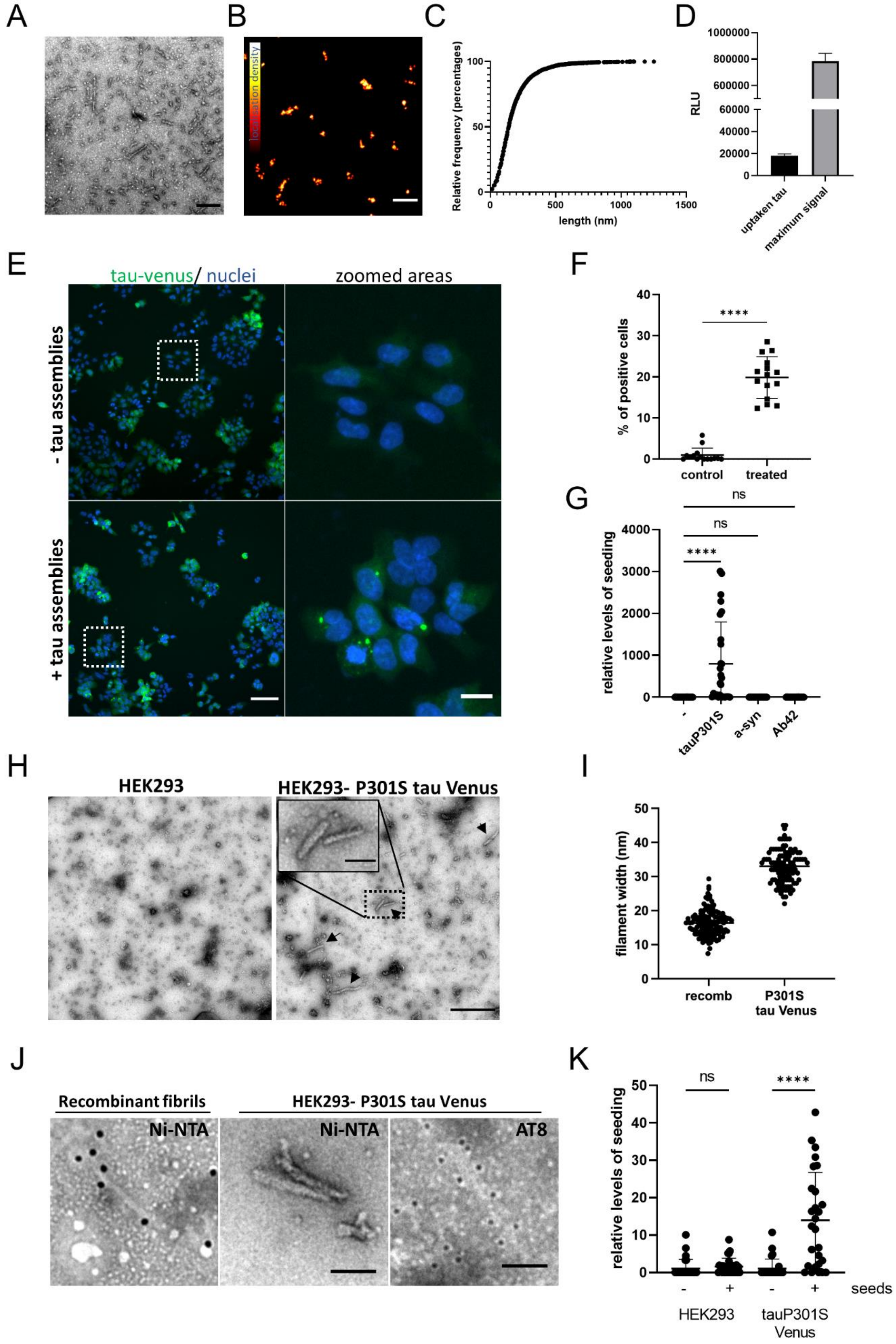

Figure S1. Characterization of heparin-assembled recombinant P301S tau fibrils, their entry to the cytosol and seeding potency (related to Figure 1). (A) Representative TEM images of sonicated heparin-assembled recombinant P301S tau fibrils. Scale bar, 200 nm. **(B)** Representative super-resolution image (scale bar, 500 nm) and **(C)** cumulative length distributions histogram as detected by DNA PAINT. **(D)** Luminescence signal of 100 nM Tau-HiBiT assemblies incubated with HEK293 cells expressing NLS-EGFP-LgBiT in the presence of lipofectamine for 24 hours (cytosolic tau) or with excess of recombinant LgBiT *in vitro* for 30 min. (n = 3 biological replicates) **(E)** Diffraction limited images of HEK293 cells stably expressing P301S tau-Venus mock-treated or treated with 100 nM heparin-assembled recombinant P301S tau fibrils. Fixed cells were imaged using the Venus fluorescence (green) for the imaging of total tau and Hoechst (blue) was used for the imaging of the nuclei. Representative zoomed in areas as indicated by the dotted boxes are shown on the right. Scale bar, 100 μm and 20 μm from left to right. **(F)** The ratio of cells positive for tau assemblies was quantified and plotted. (n > 15 FOV per condition were imaged) (n = 3 biological replicates) **(G)** The levels of seeding for HEK293 cells stably expressing P301S tau-Venus were compared to mock-treated cells upon treatment with 100 nM of recombinantly produced aggregates of a-synuclein, Aβ42 or tau (n ≥ 26,000 cells/condition analysed from 3 biological replicates). **(H)** Representative TEM images of Sarkosyl-insoluble material from control HEK293 cells and HEK293 cells expressing P301S tau-Venus that were treated with 100 nM heparin-assembled recombinant P301S tau fibrils for 24 h. The newly formed tau fibrils as a result of intracellular templated seeded reactions are highlighted with arrows. Scale bar, 100 nm and 500 nm from top to bottom. **(I)** The width of fibrils recombinantly produced or extracted from seeded cells expressing P301S tau-Venus were measured and plotted (n ≥ 100 fibrils analysed). **(J)** Representative immunogold labelling images of the recombinantly produced and the cell-extracted filaments using the Ni-NTA gold and the AT8 phospho-specific antibody (scale bar, 100 nm). **(K)** The sarkosyl-extracted material from seeded and unseeded control or P301S tau-Venus cells (0.1μl material/condition) was used for second generation seeding experiments. The relative levels of seeding were compared to unseeded control cells (n ≥ 33,000 cells/condition analysed from 3 biological replicates). All plotted data represent mean values ± SD. For statistical analysis between 2 groups an unpaired t test with Welch's correction was used, while differences between multiple means were tested by one-way ANOVA, followed by Tukey's post hoc test (n.s.= not significant, ****p < 0.0001)

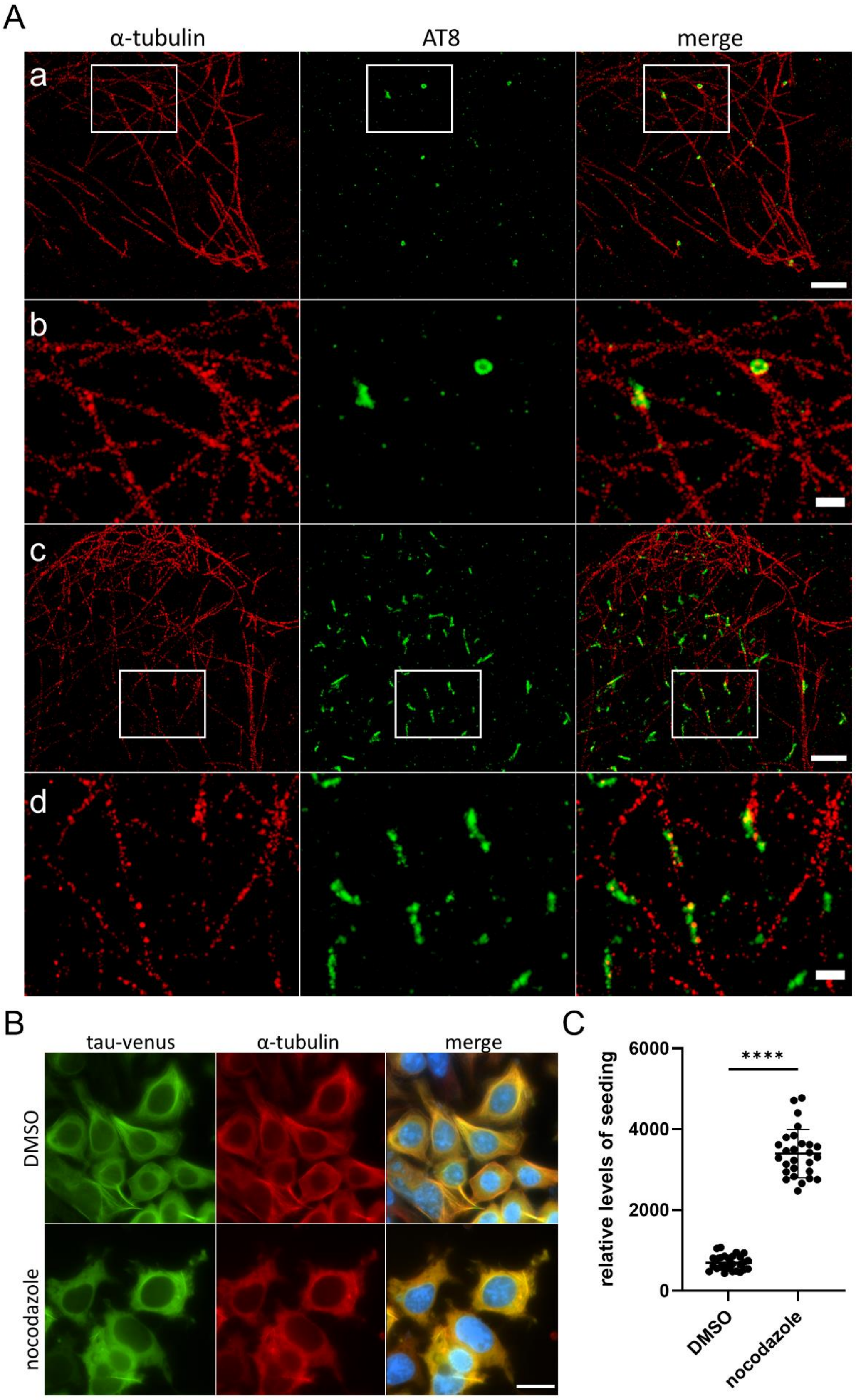

Figure S2. Tau aggregates colocalize with the microtubules as shown by Exchange DNA PAINT (related to Figure 1). (A) HEK293 P301S tau-Venus expressing cells were treated with 50 nM heparin-assembled recombinant P301S tau fibrils for 24 h. Images were obtained in two steps: first DNA-PAINT of tau aggregates

(AT8 antibody, Atto655 imager, shown in green) and then microtubules (anti-α-tubulin antibody, Atto655 imager, shown in red). Examples of 2 independent cells with lower **(a)** and higher **(c)** amounts of intracellular tau aggregates are shown. **(b)** Zoomed in area from A, as shown in box. **(d)** Zoomed in area from C, as shown in box. Scale bars represent 2 μm and 500 nm in the original (a,c) and the zoomed in areas (b,d) of each cell, respectively. **(B)** Diffraction limited images of HEK293 cells stably expressing P301S tau-Venus mock-treated or treated with 0.25 μM nocodazole for 24 hours. Fixed cells were imaged using the Venus fluorescence (green) for the imaging of total tau and tubulin (red). Scale bar, 20 μm. **(C)** The treated or untreated cells were seeded with 100 nM tau fibrils and the levels of seeding where quantified and plotted. The plotted data represent mean values ± SD. An unpaired t test with Welch's correction was used for statistical analysis (****$p < 0.0001$) ($n \geq 14,000$ cells per condition were imaged, 3 biological replicates).

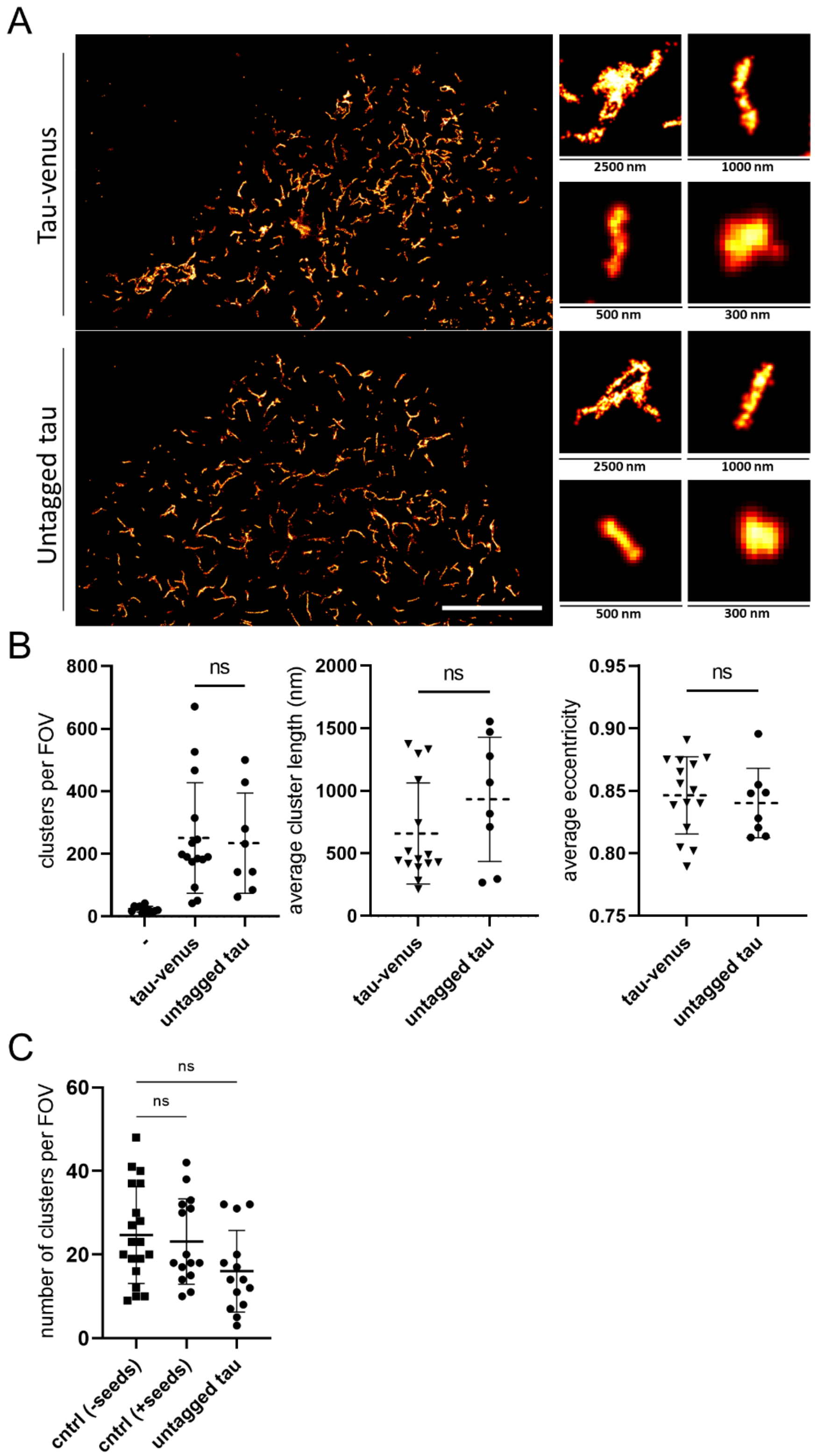

Figure S3. Comparing the shape of aggregates for the tagged and untagged P301S tau (related to Figure 1 and 2). (A) HEK293 cells expressing P301S tau-Venus or untagged P301S tau were stained using the AT8 antibody imaged by dSTORM microscopy after treatment with 100 nM heparin-assembled recombinant P301S tau fibrils for 24 h. Scale bar, 10 µm. **(B)** The number of assemblies formed, the average cluster length and the eccentricity of the formed assemblies were compared. (*n* > 8 cells per condition were imaged, 3 biological replicates, part of the data derive from Fig. 1) **(C)** Untreated P301S tau-expressing HEK293 cells (untagged tau) and control HEK293 cells (cntrl) either untreated or treated with 100 nM recombinant tau fibrils for 24h were compared for the number of detected assemblies. (*n* > 14 cells per condition were imaged, 3 biological

replicates). The plotted data represent mean values ± SD. Statistical analysis between 2 groups was performed with unpaired t test with Welch's correction, while differences between multiple means were tested by one-way ANOVA, followed by Tukey's post hoc test (n.s.= not significant)

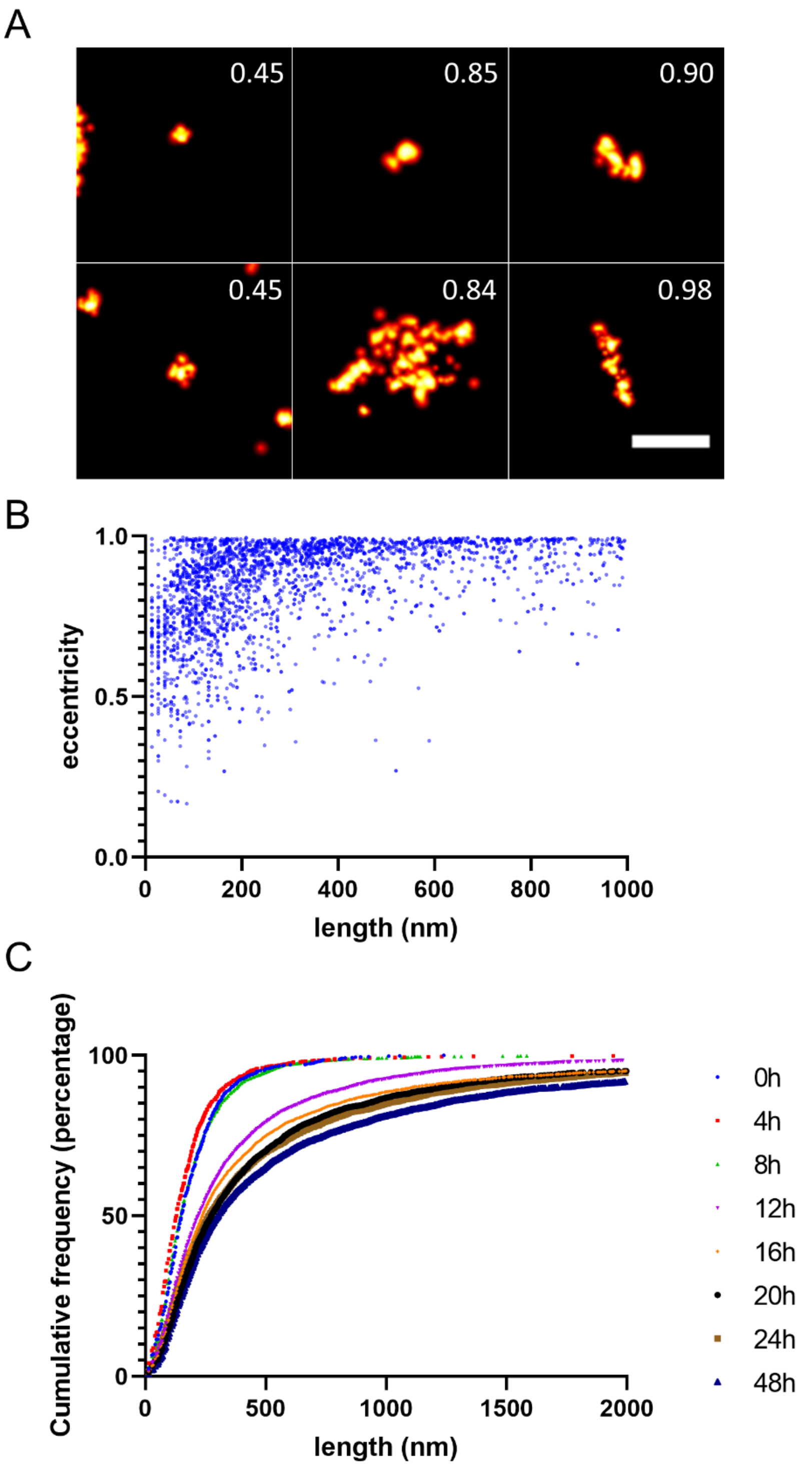

Figure S4. Eccentricity analysis of the formed P301S tau aggregates in HEK293 cells (related to Figure 2) (A) Representative examples of individual tau assemblies and their calculated eccentricity. The assemblies derive from images collected 24 h after seeding of HEK293 cells expressing untagged P301S tau. Scale bar, 500 nm. **(B)** Scatter plot of individual tau assemblies derived from images collected 24 h after seeding of HEK293 cells expressing untagged P301S tau. **(C)** Cumulative frequency distribution of the tau assemblies for the different time points.

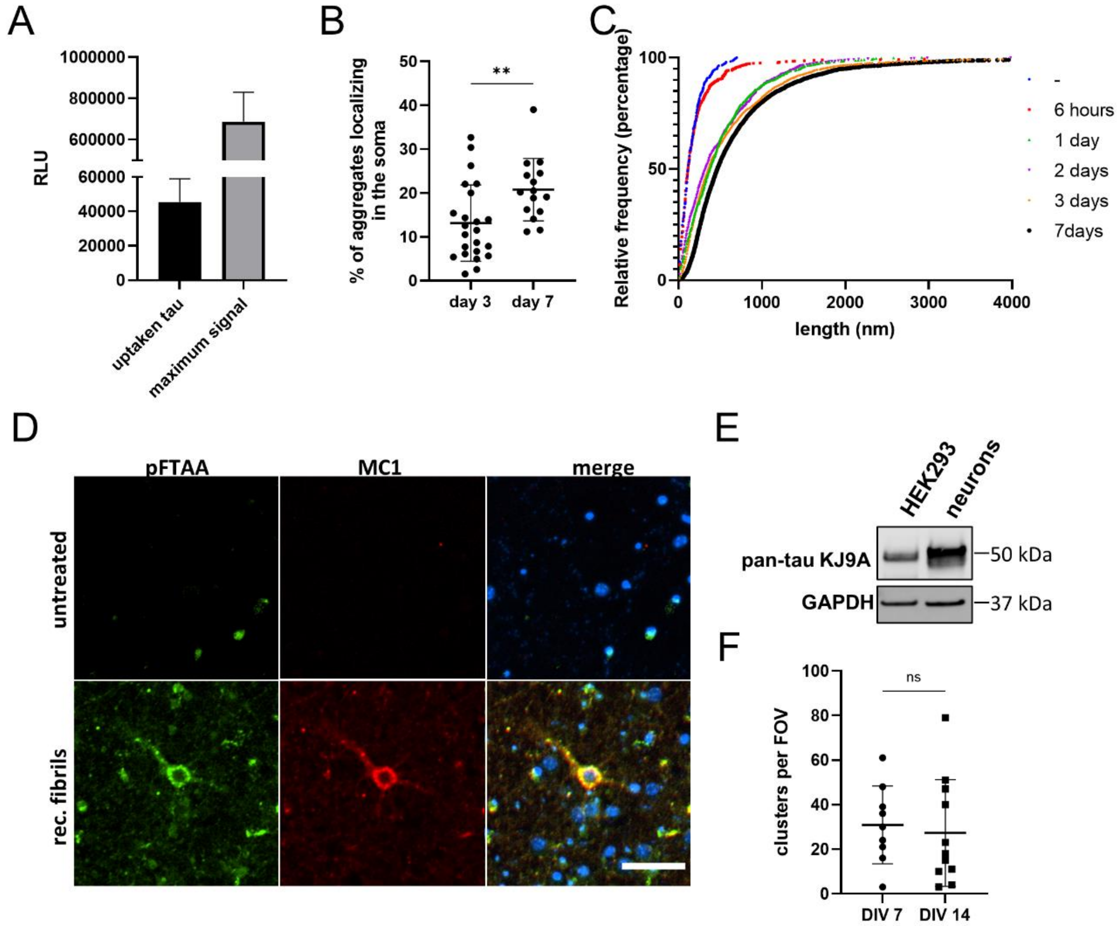

Figure S5. Seeding characterisation in cultures derived from murine primary neurons (related to Figure 3 and 4). (A) Entry of tau to the cytosol of primary neurons. Luminescence signal of 100 nM Tau-HiBiT assemblies incubated with primary cultures infected at DIV 2 with AAV1/2 hSyn::-eGFP-P2A-LgBiT-nl for 24 hours (cytosolic tau) or with excess of recombinant LgBiT *in vitro* for 30 min. (n = 4 biological replicates). **(B)** The percentage of tau assemblies detected in the soma of the neurons was quantified and plotted. **(C)** Cumulative frequency distribution of the tau assemblies for the different time points. **(D)** Diffraction limited images of P301S tau-expressing primary neurons mock-treated or treated with 100 nM heparin-assembled recombinant P301S tau fibrils for 7 days. Fixed cells were stained using pFTAA (green) and the MC1 antibody (red), while Hoechst (blue) was used for the imaging of the nuclei (scale bar, 50 nm). **(E)** WB analysis on lysates from HEK293 cells and primary cultures expressing untagged P301S tau. The cell lysates were assessed for levels of expressed tau using the pan-tau KJ9A antibody and GAPDH was employed as loading control. **(F)** 7 and 14 DIV primary cultures derived from non-transgenic mice that do not express human tau were fixed and immunostained with the AT8 antibody for dSTORM imaging. The number of assemblies detected per field of view were plotted. The statistical analysis was based on an unpaired t test with Welch's correction (n.s. not significant, $^{**}p < 0.01$) (n > 9 fields of view per condition were imaged from 2 biological replicates).

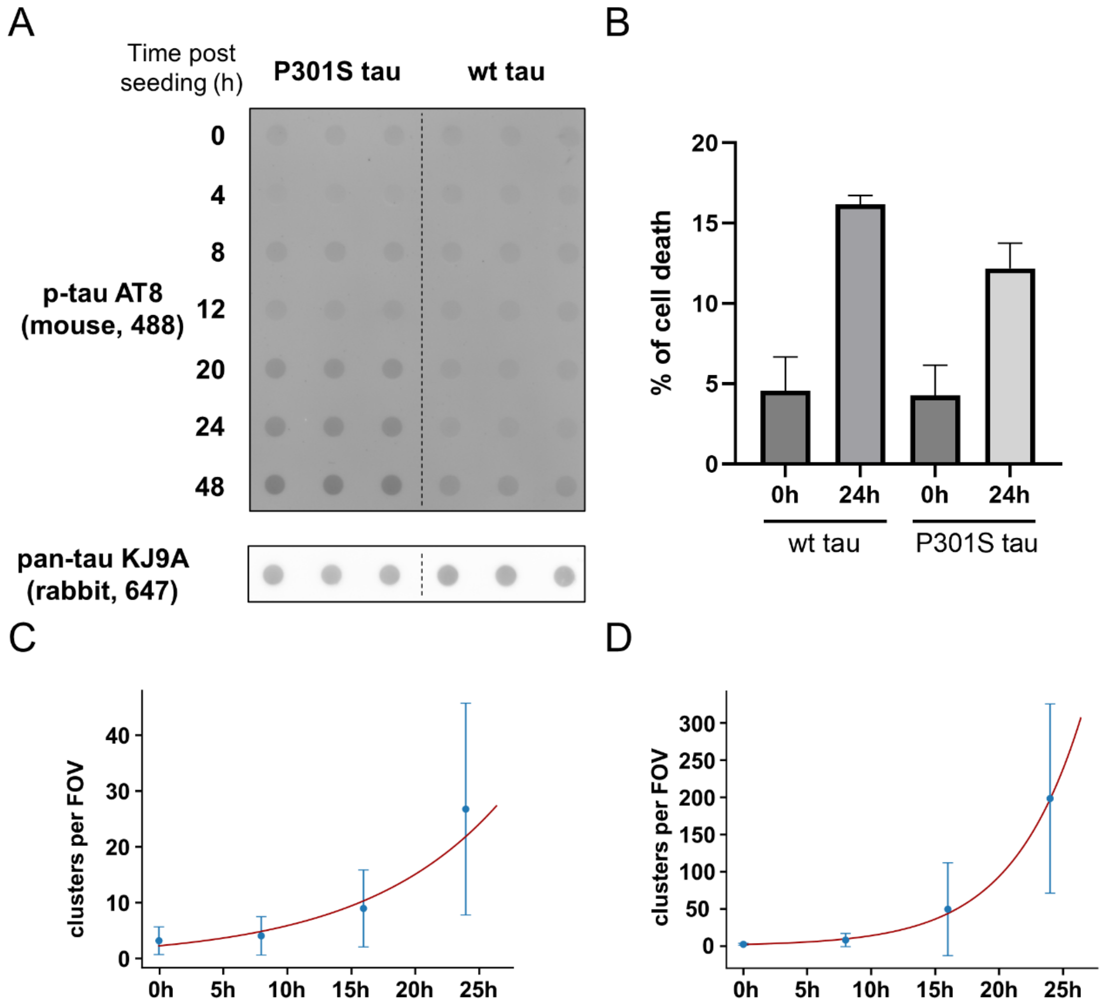

Figure S6. Expression, release and seeding kinetics comparison between HEK293 cells expressing wt and P301S tau (related to Figure 6). (A) AT8 immuno-staining on lysates from HEK293 cells expressing untagged P301S tau or wt tau upon treatment with 100 nM heparin-assembled recombinant P301S tau fibrils for different time intervals. The cell lysates collected at each time point were dotted on nitrocellulose membrane and subsequently assessed for levels of phosphorylated tau, while the total amount of tau protein was detected using the pan-tau KJ9A antibody. **(B)** Conditioned media from cells expressing wt or P301S tau variants were collected before and after the treatment with 100 nM heparin-assembled recombinant P301S tau fibrils and tested for levels of cell death, as determined by the release of LDH. Data represent mean values ± SD from 3 biological replicates. **(C, D)** Kinetic analysis of the formation of intracellular aggregates for the wt (C) and P301S (D) tau; data are shown as mean values (± SD) from Figure 6B, but all data points are used in the fitting to a minimal model of replication (see “Methods” for details).

A

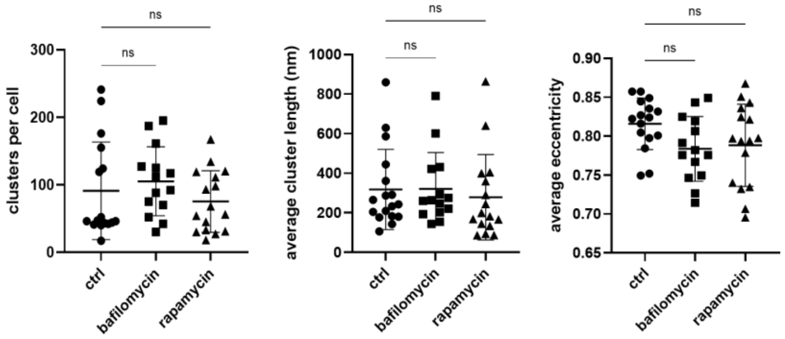

Figure S7. Autophagy inhibition or induction does not affect the templated seeded aggregation of tau in HEK293 cells (related to Figure 7). (A) HEK293 cells expressing P301S tau were treated with 50 nM heparin-assembled recombinant P301S tau fibrils in the presence of 200 nM Bafilomycin A1 or 100 nM rapamycin for 16h. The levels of aggregated tau were detected by STORM using the AT8 antibody. The number of aggregates per cell, the average length and eccentricity of the clusters were quantified. The plotted data represent mean values ± SD. The statistical analysis is based on a one-way ANOVA test combined with Tukey's post hoc test (n.s. not significant) (*n* ≥ 14 cells per condition were imaged, 3 biological replicates).

Table S1. Maximum a posteriori probability and 95% Confidence intervals of replication rate, for the different datasets (related to Figure 2, 3, 4, and Figure S6).

dataset	Max probability	95%CI lower	95%CI upper
**fixed HEK**	0.18	0.08	0.36
**HEK lysed P301S**	0.19	0.17	0.23
**HEK lysed WT**	0.09	0.06	0.14
**seeded primary neurons**	0.03	0.02	0.04
**unseeded primary neurons**	0.03	0.01	not bounded

Table S2. Calculations of replication rate and doubling time from data presented in Manos et al.[S1] (related to Figure 2, 3, 4, and Figure S6)

seed	Number @ 4 wks	Number @ 7 wks	replication rate	doubling time / days
0.016	33	33	N/A	N/A
0.030	29	61	0.04	**19**
0.063	37	111	0.05	**13**
0.123	45	180	0.07	**10**
0.250	53	270	0.08	**9**
0.499	86	389	0.07	**10**
0.993	127	529	0.07	**10**
2.021	171	627	0.06	**11**
4.023	267	742	0.05	**14**

Supplemental References

S1. Manos, J.D., Preiss, C.N., Venkat, N., Tamm, J., Reinhardt, P., Kwon, T., Wu, J., Winter, A.D., Jahn, T.R., Yanamandra, K., et al. (2022). Uncovering specificity of endogenous TAU aggregation in a human iPSC-neuron TAU seeding model. iScience *25*, 103658. 10.1016/j.isci.2021.103658.
